# Resistance to anticancer vaccination effect is controlled by a cancer cell-autonomous phenotype that disrupts immunogenic phagocytic removal

**DOI:** 10.18632/oncotarget.4754

**Published:** 2015-07-23

**Authors:** Abhishek D. Garg, Sanne Elsen, Dmitri V. Krysko, Peter Vandenabeele, Peter de Witte, Patrizia Agostinis

**Affiliations:** ^1^ Cell Death Research & Therapy (CDRT) Unit, Department of Cellular and Molecular Medicine, KU Leuven University of Leuven, Leuven, Belgium; ^2^ Laboratory for Molecular Biodiscovery, Department of Pharmaceutical Sciences, KU Leuven, Leuven, Belgium; ^3^ Molecular Signaling and Cell Death Unit, Department for Molecular Biomedical Research, VIB, Ghent, Belgium; ^4^ Department of Biomedical Molecular Biology, Ghent University, Ghent, Belgium

**Keywords:** immunogenic cell death, calreticulin, patient, predictive biomarker, prognostic biomarker

## Abstract

Immunogenic cell death (ICD) is a well-established instigator of ‘anti-cancer vaccination-effect (AVE)’. ICD has shown considerable preclinical promise, yet there remain subset of cancer patients that fail to respond to clinically-applied ICD inducers. Non-responsiveness to ICD inducers could be explained by the existence of cancer cell-autonomous, anti-AVE resistance mechanisms. However such resistance mechanisms remain poorly investigated. In this study, we have characterized for the first time, a naturally-occurring preclinical cancer model (AY27) that exhibits intrinsic anti-AVE resistance despite treatment with ICD inducers like mitoxantrone or hypericin-photodynamic therapy. Further mechanistic analysis revealed that this anti-AVE resistance was associated with a defect in exposing the important ‘eat me’ danger signal, surface-calreticulin (ecto-CRT/*CALR*). In an ICD setting, this defective ecto-CRT further correlated with severely reduced phagocytic clearance of AY27 cells as well as the failure of these cells to activate AVE. Defective ecto-CRT in response to ICD induction was a result of low endogenous CRT protein levels (i.e. CRT^low^-phenotype) in AY27 cells. Exogenous reconstitution of ecto-rCRT (recombinant-CRT) improved the phagocytic removal of ICD inducer-treated AY27 cells, and importantly, significantly increased their AVE-activating ability. Moreover, we found that a subset of cancer patients of various cancer-types indeed possessed *CALR*^low^ or CRT^low^-tumours. Remarkably, we found that tumoural *CALR*^high^-phenotype was predictive of positive clinical responses to therapy with ICD inducers (radiotherapy and paclitaxel) in lung and ovarian cancer patients, respectively. Furthermore, only in the ICD clinical setting, tumoural *CALR* levels positively correlated with the levels of various phagocytosis-associated genes relevant for phagosome maturation or processing. Thus, we reveal the existence of a cancer cell-autonomous, anti-AVE or anti-ICD resistance mechanism that has profound clinical implications for anticancer immunotherapy and cancer predictive biomarker analysis.

## INTRODUCTION

The field of anticancer immunotherapies encompassing “active” cell-based vaccines has experienced an unprecedented level of development in recent times [1]. For most cell-based anticancer vaccines, “whole cancer cells” (induced to undergo cell death) represent the central element [2]. Thus not surprisingly, a significant effort has also been invested in increasing the immunogenicity of dying cancer cells used for vaccine production [3–5]. The field has rapidly moved from simple induction of freeze-thawing-based necrosis (which is not highly immunogenic) to more sophisticated methodologies for increasing the “whole cancer cell” immunogenicity [5, 6]. One such emerging methodology is ‘immunogenic cell death (ICD)’ [7–9].

ICD exhibits high immunogenicity owing to the spatiotemporally-defined emission of damage-associated molecular patterns (DAMPs) capable of acting as immunogenic danger signals [7–9]. Such DAMPs include the surface exposed (ecto-), endoplasmic reticulum (ER) chaperone, calreticulin (CRT), secreted ATP and post-apoptotic released molecules like high-mobility group box-1 (HMGB1) protein [7–10]. Amongst these DAMPs, the immunogenic ‘eat me’ signal, ecto-CRT tends to exclusively associate with ICD [6, 11–14]. In an operational sense, ICD activates ‘anti-cancer vaccination effect (AVE), i.e., cancer cells undergoing ICD *in vitro* and administered *in vivo* are capable of eliciting potent tumour-rejecting immunity (demonstrated in number of mice models) [7]. Moreover, tumour cells undergoing ICD *in vivo* can also activate an “*in situ*” AVE that “primes” the immune system to specifically eliminate residual cancer cells [7, 15].

It is noteworthy that, several ICD inducers have been applied for clinical treatment of cancer patients, either as experimental therapies (e.g. Hypericin-based Photodynamic Therapy/Hyp-PDT) or as standards-of-care and palliative-care therapies (e.g. radiotherapy, mitoxantrone, paclitaxel) [1, 7, 9]. However, despite treatment with ICD inducers, there remains a subset of patients showing refractoriness to therapy [16–18]. Considering the strong preclinical evidence of ICD-elicited immunity, it is possible that the above subset of patients exhibit poor prognosis at least partially due to the failure of anti-tumour immunity. Such resistance mechanisms need to be urgently characterized since they represent major stumbling blocks for the clinical success of ICD.

So far, the major naturally-occurring anti-ICD or anti-AVE resistance mechanisms have been described on the level of host immune system (e.g. mutations in immune-receptors that disrupt immunogenicity-sensing) [4, 8, 15]. However, considering the centrality occupied by cancer cells in the vaccination set-up, it is absolutely vital to uncover cancer cell-autonomous mechanisms that reduce immunogenicity – an area seldom investigated. Of note, our recent meta-analysis advocated the existence of cancer cell-autonomous mechanisms ablating immunogenic danger signalling [4, 8]. The impact that such resistance mechanisms can have, has been confirmed mostly through synthetic gene-knockdown/knockout strategies (e.g. RNAi) [6, 12, 14, 15]. However, such synthetic strategies either harbour the risk of off-target effects or are not representative of intrinsic mechanisms [19]. Thus the existence of a naturally-occurring experimental model exhibiting intrinsic anti-AVE or anti-ICD resistance would be critical. Such a model could lead to a better understanding of similar resistance mechanisms in a subset of patients showing poor prognosis following treatment with ICD inducers.

While broad resistance mechanisms not affected by ICD inducer-to-inducer differences may exist yet it has been proposed that Hyp-PDT might be comparatively less susceptible to various resistance mechanisms compared to chemotherapy or radiotherapy [4, 20, 21]. Owing to this we envisaged that an experimental model showing *in vivo* resistance to Hyp-PDT treatment has the possibility of exhibiting the broadest possible AVE-resistant phenotype. To this end, we did a literature survey and found one such experimental model that fitted this criteria i.e. AY27 rat bladder cancer model [22, 23]. Previous studies showed that established AY27 tumours in rats exhibited strong initial responses to Hyp-PDT treatment, characterized by massive tumour-debulking. However, 1–3 weeks after treatment, these tumours relapsed thus indicating their refractoriness to Hyp-PDT treatment [22, 23]. This observation stands in stark contrast to the well-established ability of Hyp-PDT to induce *bona fide* ICD, AVE and robust anti-tumour immunity [6, 12, 13, 24, 25] e.g. treatment of established CT26 tumours [9] in mice with Hyp-PDT was associated with 100% eradication of these tumours and not accompanied by relapse, such that even re-challenge of these mice with live CT26 cells prevented new tumour growth [9, 25]. As a whole this suggests that through as-yet-unknown phenomena, AY27 cancer cells display the ability to resist the action of a *bona fide* ICD inducer thereby making it an interesting experimental model for studying anticancer vaccination resistance.

To this end, the primary aim of this study was to investigate whether AY27 is a naturally-occurring experimental model of intrinsic resistance to AVE. Furthermore, we wished to uncover the mechanism underlying this resistance (i.e. ICD based or not). We also aimed to investigate, through retrospective meta-analysis of publicly available datasets, whether subset of cancer patients may exhibit similar disparity. Finally, we wanted to ascertain whether the above characterized mechanisms of AVE resistance may serve as a ‘predictive biomarker(s)’ of the efficacy of ICD inducers in clinical settings.

## RESULTS

### Rat bladder cancer AY27 cells exhibit intrinsic resistance to ‘anticancer vaccination effect’

**Figure 1:**
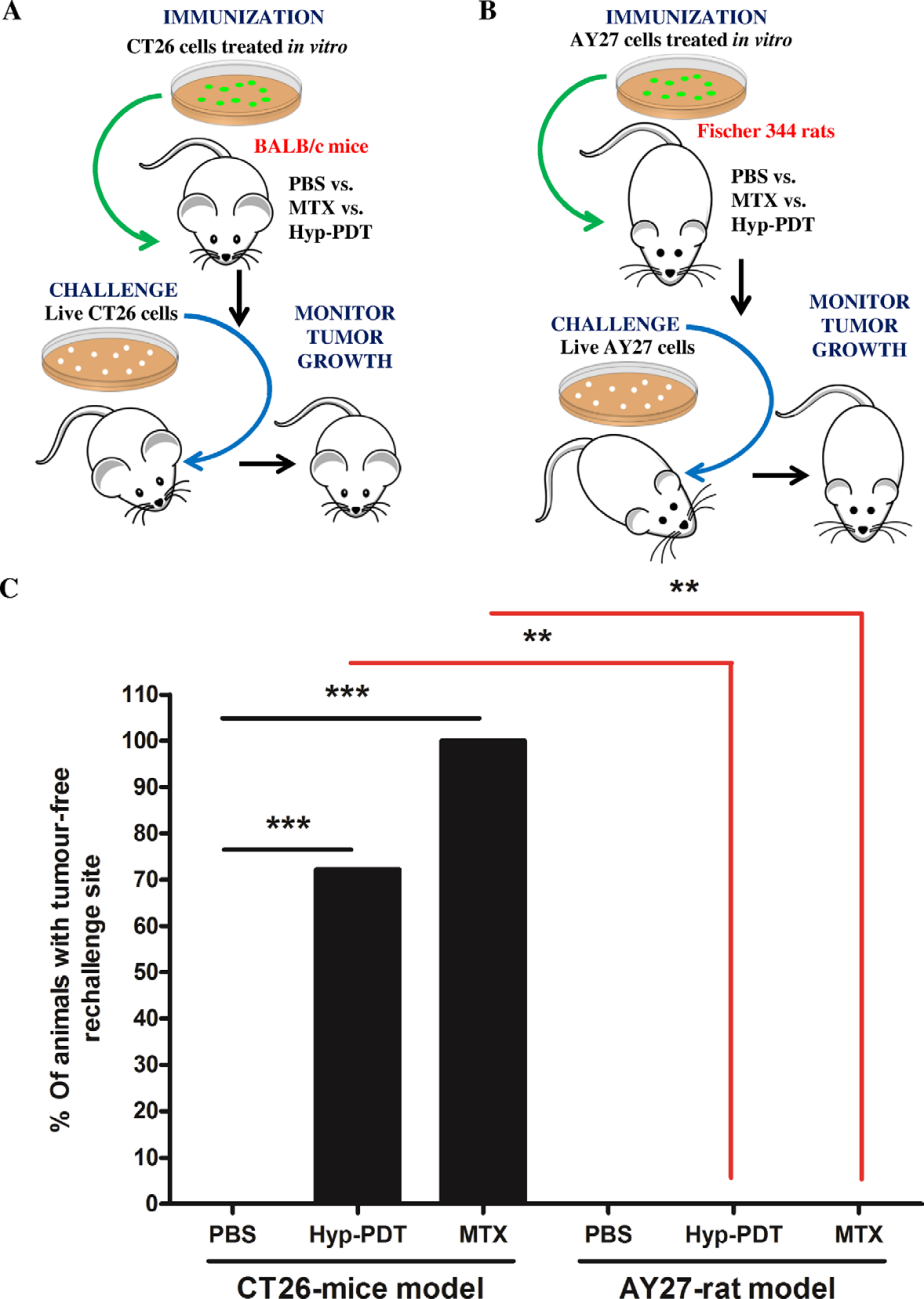
Rat bladder carcinoma AY27 cells exhibit resistance to ‘anticancer vaccination effect’ associated with ICD inducers CT26 cells **A.** or AY27 cells **B.** were treated *in vitro* with Hyp-PDT (150 nM Hyp incubated for 16 h followed by irradiation with light fluence of 2.70 J/cm^2^) or mitoxantrone (MTX; 1 μM), followed by recovery at 24 h post-treatment. These treated CT26 and AY27 cells were then injected subcutaneously into BALB/c mice (PBS, *n* = 10 mice; Hyp-PDT, *n* = 18 mice; MTX, *n* = 6 mice) and Fischer 344 rats (PBS, *n* = 6 rats; Hyp-PDT, *n* = 6 rats; MTX, *n* = 6 rats), respectively. Eight to ten days post-vaccination, the mice and rats were challenged in contra-lateral flank with live CT26 (A) and live AY27 (B) cells, respectively. Mice or rats injected with PBS were utilized as placebo-controls. **C.** This was followed by monitoring of tumour incidence at the challenge site. Statistical analysis was performed using the Fischer's exact test; statistical significance between conditions is indicated by the bars (*p < 0.05, **p < 0.01, ***p < 0.0001).

Based on the findings showing AY27-tumor's tendency to relapse despite treatment with the prototypical ICD-inducing agent, Hyp-PDT [22, 23]; we decided to examine whether this failure was due to the AY27 cells' inability to activate AVE. In absence of a *bona fide* ICD-susceptible rat cancer model, for comparative purposes, we used the CT26 murine cancer cells [6, 13]. CT26 cancer model is a well-established AVE/ICD-susceptible model [14, 25, 26]. We exposed both CT26 and AY27 cells to two prototypical inducers of AVE i.e. Hyp-PDT and the chemotherapeutic, mitoxantrone (MTX) for 24 h. The resulting preparations of similarly dead or dying, apoptotic, CT26 (Suppl. Fig. S1A–S1B) or AY27 cells (Suppl. Fig. S1A–S1B) were injected subcutaneously into left flank of syngeneic immune-competent BALB/c mice (Fig. 1A) and Fischer 344 rats (Fig. 1B), respectively. Post-vaccination, these rodents were re-challenged with live CT26 (Fig. 1A) or AY27 (Fig. 1B) cells as applicable, in the opposite flank(s). Thereafter, protection against tumour growth at the re-challenge site was interpreted as a sign of antitumor vaccination, as described previously [6, 13]. The ICD-susceptible CT26 cells exhibited high efficiency in activating AVE such that 70–100% BALB/c mice ‘vaccinated’ with MTX or Hyp-PDT treated CT26 cells exhibited efficient tumour-rejecting responses (Fig. 1C). In a stark contrast, none of the rats ‘vaccinated’ with MTX or Hyp-PDT treated AY27 cells exhibited tumour-rejecting responses, such that all of them developed tumours at the re-challenge site (Fig. 1C).

### Rat bladder cancer AY27 cells exhibit disruption in calreticulin surface exposure and inefficient phagocytic removal by professional phagocytes

The inability of AY27 cells treated with ICD-inducers to elicit AVE raised a possibility that perhaps these cells have some defect in ICD-associated danger signalling. To address this possibility, we decided to investigate the most important hallmarks of ICD i.e. secretion of ATP, release of HMGB1, ecto-CRT and efficient phagocytic removal by professional phagocytes.

**Figure 2:**
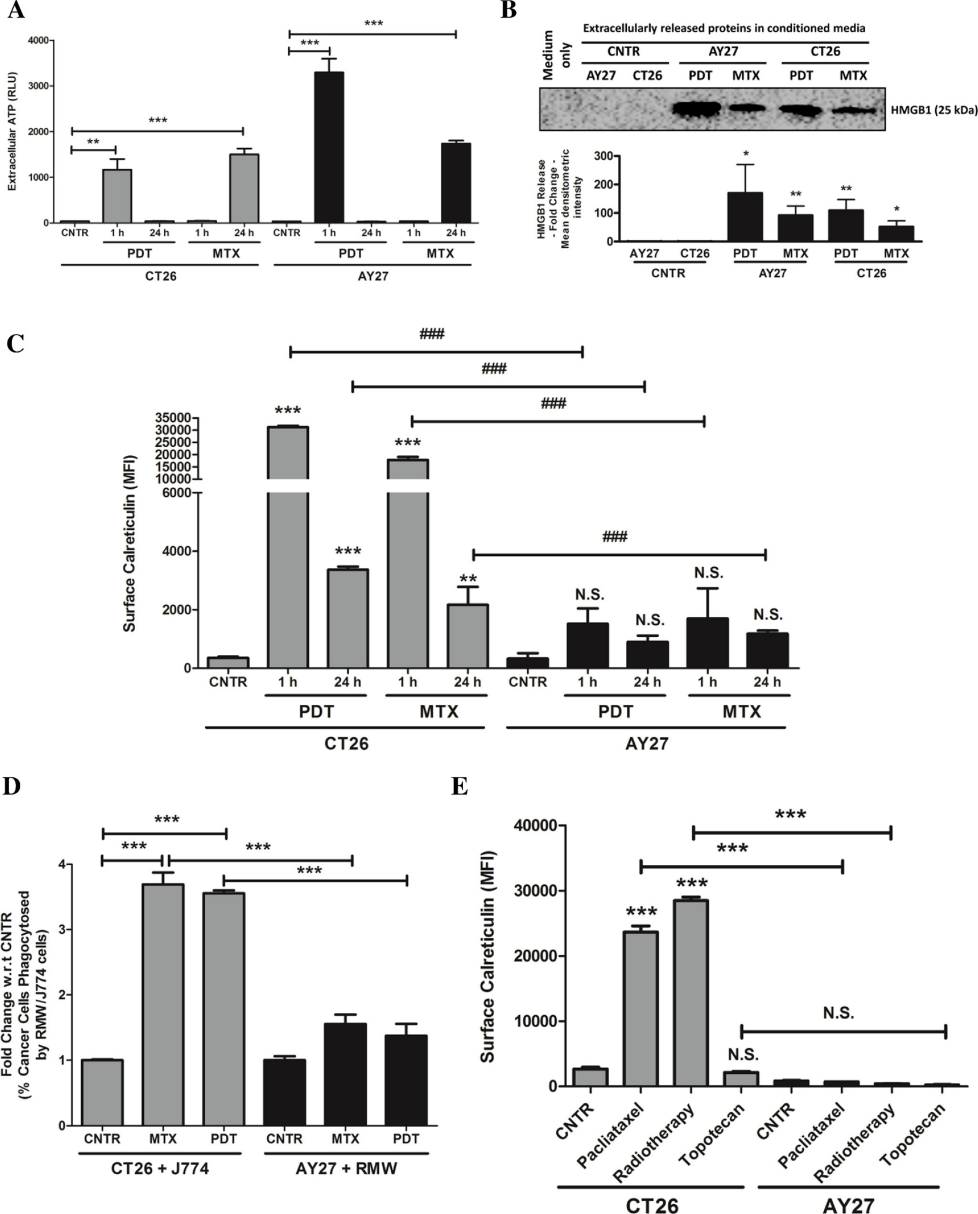
AY27 cancer cells treated with ICD inducers exhibit disruption in the ability to surface expose CRT and undergo inefficient engulfment by phagocytic cells CT26 cells or AY27 cells were treated or not (i.e. untreated controls/CNTR) with Hyp-PDT (150 nM Hyp incubated for 16 h followed by irradiation with light fluence of 2.70 J/cm^2^) or MTX (1 μM). This was followed by – **A.** analysis of extracellular ATP at indicated time points such that the data is presented as relative light units (RLU) values (*n* = 3; mean ± s.e.m.; *p < 0.05, **p < 0.01, ***p < 0.0001 as indicated by bars; Student's *t*-test); **B.** analysis of extracellularly released HMGB1 in 24 h post-treatment conditioned media via immunoblotting (a graph indicating the mean densitometry intensity of HMGB1 is presented; media-only values have been subtracted; *n* = 3; mean ± s.d.; *p < 0.05, **p < 0.01, versus respective CNTR; Student's *t*-test); **C.** and analysis for surface exposed calreticulin in non-permeabilised cells, performed at indicated time-points, such that the data is presented as mean fluorescence intensity (MFI) (*n* = 3; mean ± s.d.; *p < 0.05, **p < 0.01, ***p < 0.0001 versus respective CNTR, ^###^*p* < 0.0001 as indicated by bars and N.S = non-significant with respect to CNTR; One-way ANOVA with Dunnett's test for comparison with respective CNTR and Bonferroni's test for comparison between other conditions). **D.** In another case, the phagocytic engulfment of JADE^+^CT26 and JADE^+^AY27 cancer cells (recovered 1 h post-treatment) by NIR780^+^J774 and NIR780^+^RMW phagocytic cells, respectively, was measured after 4 h of co-incubation. Amount of cancer cells phagocytosed by phagocytes were scored by determining the percentage of double positive events in FACS analysis (i.e. NIR780^+^/JADE^+^, representing phagocytosed cancer cells; data is expressed as fold change with respect to/w.r.t. average of respective CNTR; *n* = 3; mean ± s.e.m.; *p < 0.05, **p < 0.01, ***p < 0.0001 as indicated by the bars; One-way ANOVA with Dunnett's test for comparison with respective CNTR and Bonferroni's test for comparison between other conditions). **E.** CT26 or AY27 cells were treated or not (i.e. untreated controls/CNTR) with paclitaxel (1 μM), radiotherapy (75 Gy) and topotecan (2 μM) followed by analysis for surface exposed calreticulin in non-permeabilised cells, performed at 12 h post-treatment time-point. Data is presented as mean fluorescence intensity (MFI) (*n* = 3; mean ± s.e.m.; *p < 0.05, **p < 0.01, ***p < 0.0001 and N.S = non-significant as indicated by bars; One-way ANOVA with Bonferroni's test).

Analysis of secreted ATP showed that, following treatment with MTX or Hyp-PDT, both CT26 and AY27 cancer cells exhibited increased ATP secretion (Fig. 2A). As reported previously [6, 12], Hyp-PDT treatment induced rapid ATP secretion (detectable as soon as, 1 h post-treatment) in both cell types. On the other hand, MTX-elicited ATP secretion was comparatively late (24 h post-treatment) (Fig. 2A). ATP secretion by AY27 cells after Hyp-PDT was considerably higher than in CT26 cells (Fig. 2A); whereas MTX induced similar levels of ATP secretion from both cancer cell lines (Fig. 2A). Following treatment with MTX or Hyp-PDT, both cancer cell-types exhibited increased release of HMGB1 (Fig. 2B), a DAMP associated to cell's secondary necrosis, following a trend that paralleled the accumulation of secondary necrotic cells in response to these ICD inducers (Suppl. Fig. S1A–S1B).

Next, we analysed the ability of these ICD inducers to elicit ecto-CRT. CT26 cancer cells efficiently and rapidly mobilized CRT at their surface after both MTX and Hyp-PDT treatments (detectable as soon as, 1 h post-treatment) (Fig. 2C). In stark contrast, ecto-CRT levels were strongly reduced (but not nil) in AY27 cancer cells, after treatment with both MTX and Hyp-PDT (Fig. 2C). This disparity in AY27 cells was also confirmed by cell surface protein biotinylation analysis (data not shown). As ecto-CRT serves as a potent ‘eat-me signal’, we next analysed the ability of these treated cancer cells to undergo phagocytic removal by professional phagocytes. MTX or Hyp-PDT treated CT26 cells underwent highly efficient phagocytosis by the J774 murine phagocytes [27] (Fig. 2D). In stark contrast, MTX or Hyp-PDT treated AY27 cells were barely phagocytosed by the RMW rat phagocytes [28] (Fig. 2D).

We then wondered whether failure to mobilize ecto-CRT by the AY27 cells, was a common feature of other anticancer therapies known to induce ICD, such as radiotherapy [14, 26] or paclitaxel [29]. Moreover we expanded our analysis in CT26 and AY27 cells also to a prototype of non-ICD inducers, e.g. topotecan (a camptothecin analogue) [14]. To this end, we treated CT26 and AY27 cells with the above agents and analysed ecto-CRT. Similar to the above trends, while radiotherapy and paclitaxel both efficiently induced ecto-CRT in CT26 cells (Fig. 2E) yet these ICD inducers failed to elicit similar amounts of ecto-CRT in AY27 cells (Fig. 2E). In line with its non-ICD functionality, topotecan failed to induce ecto-CRT in both CT26 and AY27 cells (Fig. 2E). In aggregate, these results indicate that, compared to CT26 cells, AY27 cells exhibit defects in ecto-CRT presentation in response to ICD inducers, which correlates with their inability to undergo efficient phagocytic removal.

### Reconstitution of exogenous calreticulin in AY27 cells increases their phagocytic removal and their ability to elicit ‘anticancer vaccination effect’

**Figure 3:**
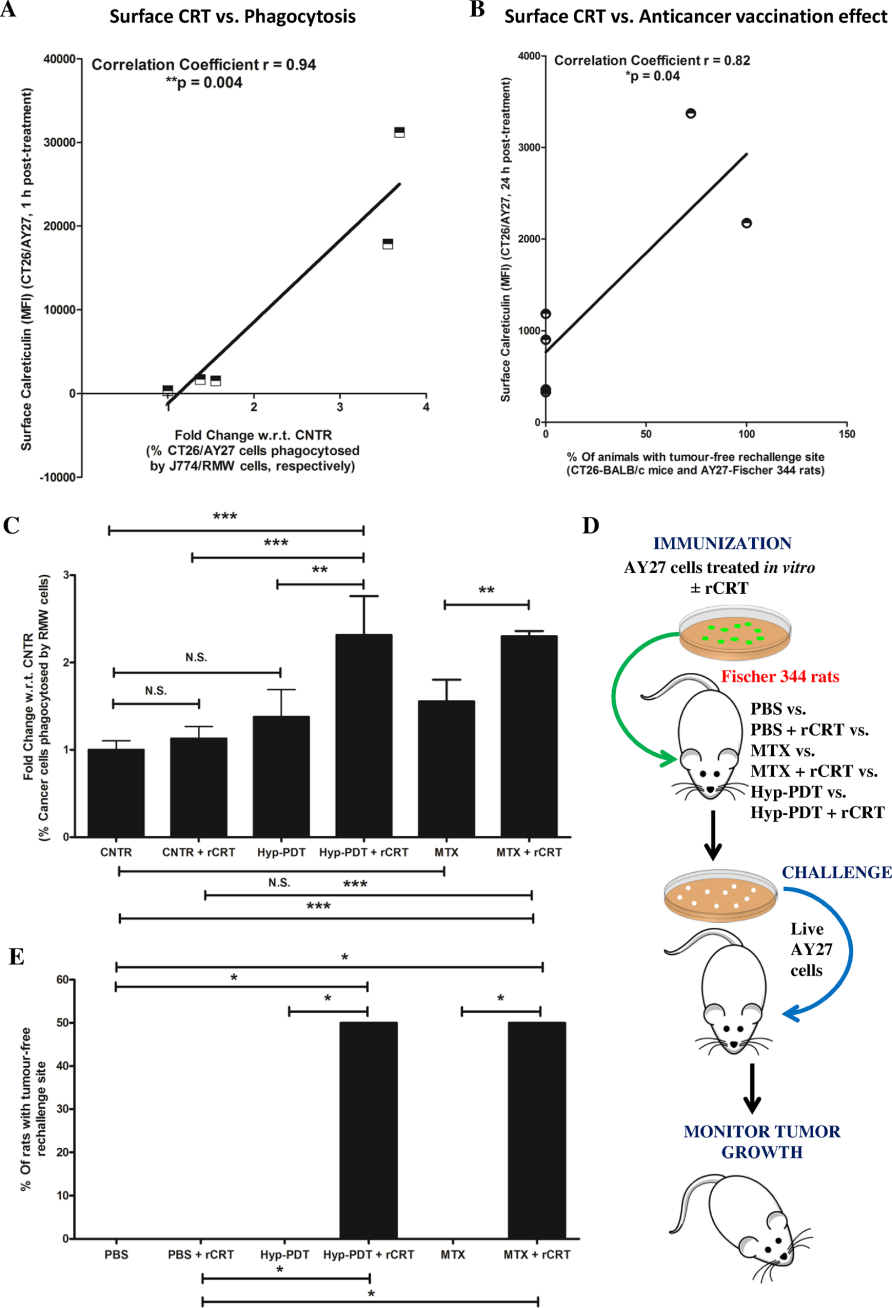
Reconstituting exogenous CRT in AY27 cancer cells, treated with ICD inducers, improves their ability to undergo phagocytosis *in vitro* and exhibit ‘anticancer vaccination effect’ A correlation analysis was carried out between the amount of surface exposed CRT as evident in Fig. 2C and either *in vitro* phagocytosis of cancer cells by innate immune cells as evident in Fig. 2D **A.** or *in vivo* anticancer vaccination effect as evident in Fig. 1C **B.** (*p < 0.05 or **p < 0.01; correlation coefficient and *p* values are mentioned on the graph; Student's *t*-test). **C–E.** Rat bladder carcinoma AY27 cells were treated or not (i.e. untreated controls/CNTR) with Hyp-PDT (150 nM Hyp incubated for 16 h followed by irradiation with light fluence of 2.70 J/cm^2^) or MTX (1 μM). **C.** Phagocytic engulfment of JADE^+^AY27 cancer cells (recovered 1 h post-treatment and first incubated, or not, with recombinant CRT (rCRT)) by NIR780^+^RMW phagocytic cells was measured after 4 h of co-incubation. Amount of cancer cells phagocytosed by phagocytes were scored by determining the percentage of double positive events in FACS analysis (i.e. NIR780+/JADE+, representing phagocytosed cancer cells; data is expressed as fold change with respect to/w.r.t. average of CNTR; *n* = 3; mean ± s.d.; *p < 0.05, **p < 0.01, ***p < 0.0001 as indicated by the bars; One-way ANOVA with Newman-Keuls test). In another case (D-E), AY27 cells treated *in vitro* with Hyp-PDT or MTX (as described above), were recovered 24 h post-treatment. These cells were incubated (or not) with rCRT for 30 min, washed and injected subcutaneously into Fischer 344 rats (PBS, *n* = 5 rats and PBS+rCRT, *n* = 5 rats; Hyp-PDT *n* = 6 rats and Hyp-PDT+rCRT, *n* = 6 rats; MTX, *n* = 6 rats and MTX+rCRT, *n* = 6 rats), respectively. Eight to ten days post-vaccination, the rats were rechallenged in contra-lateral flank with live AY27 cells **D.** Rats injected with PBS or PBS plus rCRT were utilized as placebo-controls (5 rats each). This was followed by monitoring of tumour incidence at the rechallenge site **E.** Statistical analysis was performed using the Gehan-Breslow-Wilcoxon Test; statistical significance is indicated by the bars (*p < 0.05).

A correlation analysis of the above data showed that across CT26 and AY27 systems, there was a strong, positive linear correlation (*p* < 0.05) between the levels of ecto-CRT and efficient phagocytic removal (Fig. 3A) as well as AVE-inducing capacity (Fig. 3B). This reaffirmed the activity of ecto-CRT as an ‘eat me’ signal and an immunogenicity-definer. To this end, it was imperative to confirm whether the disparity in ecto-CRT levels was behind AY27 cells' inefficient phagocytosis and resistance to AVE.

To address this, we analysed the ability of treated AY27 cells (post-incubated or not, with exogenously ‘reconstituted’ recombinant CRT/rCRT) to undergo phagocytic removal by professional phagocytes. In line with the above observations (Fig. 2D), phagocytosis of MTX or Hyp-PDT treated AY27 cells by the RMW rat phagocytes was insignificant (Fig. 3C). However, reconstitution of ecto-rCRT, statistically significantly increased the phagocytic clearance of treated AY27 cells by RMW rat phagocytes (Fig. 3C).

Next, we decided to probe the possible functional link between the lack of ecto-CRT and AVE-resistance in AY27 cells. To this end, we exposed AY27 cells to Hyp-PDT and MTX for 24 h; and the resulting mixture of dead and dying AY27 cells was incubated or not, with exogenously ‘reconstituted’ ecto-rCRT followed by their injection into Fischer 344 rats. This was followed by re-challenging with live AY27 cells in the contra-lateral flank (Fig. 3D). In line with the above observations (Fig. 1C), rats ‘vaccinated’ with MTX or Hyp-PDT treated AY27 cells again failed to reject AY27 tumours at the re-challenge site (Fig. 3E). However interestingly, injection with vaccines reconstituted with ecto-rCRT, significantly increased the number of rats (by approximately 50%) capable of resisting AY27 tumour growth at the re-challenge site (Fig. 3E). The latter results establish low ecto-CRT as a chief defect behind AY27 cell's inability to activate AVE.

### Low endogenous levels of calreticulin cause disruption in calreticulin surface exposure capacity

Ecto-CRT exposure is mediated by a multi-factorial signalling pathway [4, 8, 30]. In case of MTX, secondary non-lethal ER stress activation and caspases activity are required for ecto-CRT [4, 8, 30]. On the other hand, Hyp-PDT–induced ecto-CRT, requires lethal ER stress but is not reliant on caspase activation [4, 6, 8, 31]. Thus, it was imperative to characterize whether these broad signalling events were intact in AY27 cells.

Analysis of the apoptotic module showed that both MTX and Hyp-PDT induced increase in the amount of cleaved caspase-3 and downstream cleavage of PARP (molecular markers of intrinsic apoptosis) with similar efficiency in both cancer cell-types (Suppl. Fig. S2A). Analysis of ER stress module showed that relative to CT26 cells, ER stress responses (marked by eIF2α phosphorylation, Suppl. Fig. S2B; BiP/GRP78 up-regulation and CHOP activation, Suppl. Fig. S2C) were not reduced in AY27 cancer cells but were even accentuated to a certain extent (especially in case of eIF2α phosphorylation, Suppl. Fig. S2B), thus indicating that activation of the apoptotic module and ER stress was intact in both cancer cell-types. Moreover, inhibition of caspases signalling through the pan-caspase inhibitor, zVAD-fmk [32], and attenuation of ER stress through a well-characterized chemical chaperone, TUDCA [32], improved cell survival after Hyp-PDT treatment, in both cancer cell-types (Suppl. Fig. S3). In contrast, only pre-treatment with zVAD-fmk, but not TUDCA, improved cellular survival after MTX treatment for both cancer cell-types (Suppl. Fig. S3). Overall, these results clearly show that the activation and signalling functions of ER stress, which is mandatory for ecto-CRT emission, is intact in the AY27 cells. Of note, these results are substantiated by our recent observations of morphological ER stress signatures in Hyp-PDT treated AY27 tumours, *in vivo* [33].

**Figure 4:**
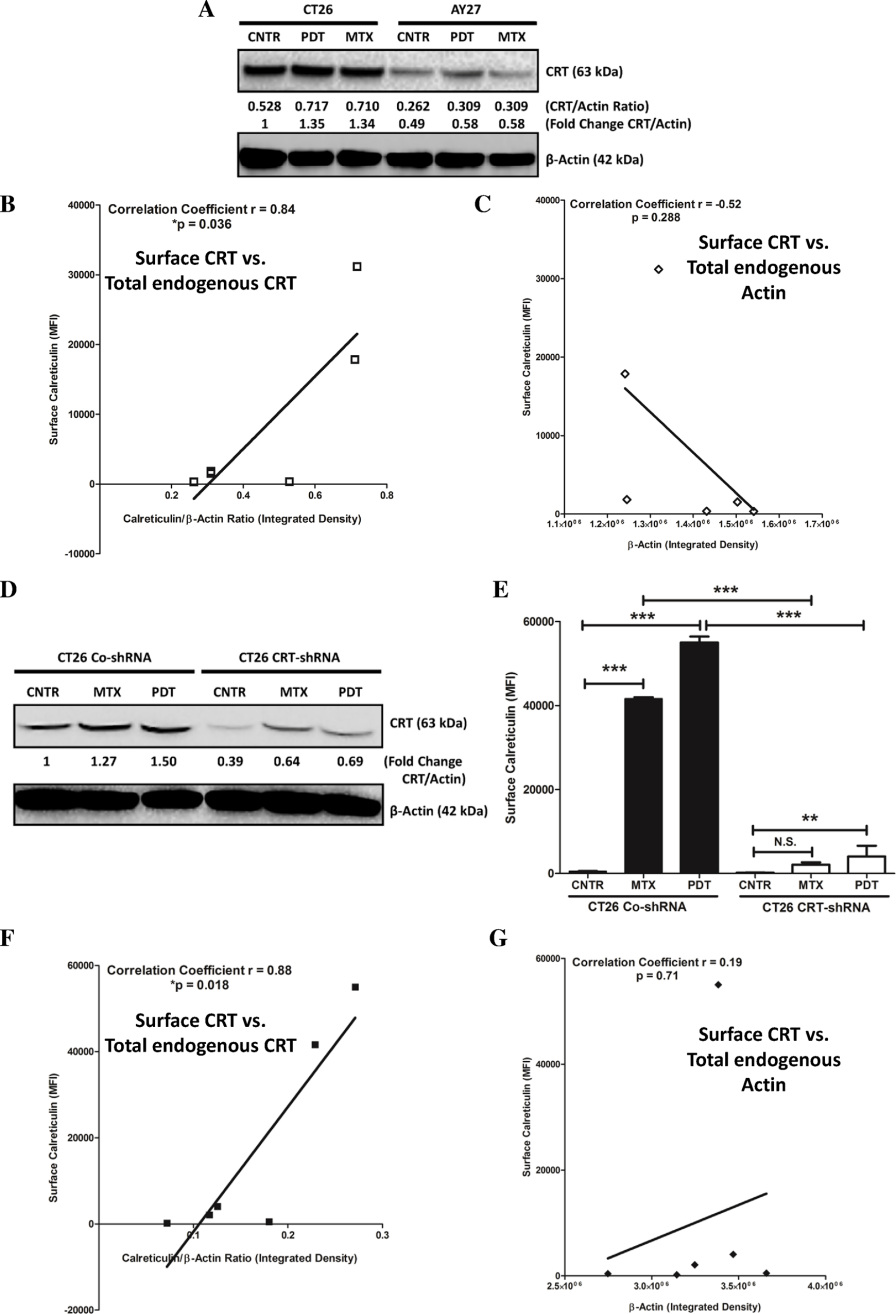
AY27 cancer cells exhibit low endogenous levels of CRT which are associated with a proportionally low surface exposure of CRT in response to ICD inducers CT26 cells or AY27 cells were treated or not (i.e. untreated controls/CNTR) with Hyp-PDT (150 nM Hyp incubated for 16 h followed by irradiation with light fluence of 2.70 J/cm^2^) or MTX (1 μM). This was followed by immunoblotting analysis for CRT, 1 h post-treatment **A.** The calculations based on band densitometry analysis are mentioned as applicable i.e. ratio of endogenous levels of CRT to Actin and the fold-change in this ratio relative to CNTR. Moreover, the bands for CRT and Actin in **A** for all the conditions were quantified for their integrated density followed by correlation analysis between the amount of surface exposed calreticulin (as evident in Fig. 2C) and endogenous total CRT levels **B.** or endogenous total Actin levels **C.** (*p < 0.05; correlation coefficient and *p* value are mentioned on the graph; Student's *t*-test). Next, we stably transfected CT26 cancer cells with control-shRNA (CO-shRNA) or shRNA targeting CRT (CRT-shRNA) and the respective clones thus obtained were treated (or not) as described above. This was followed by immunoblotting analysis for CRT, 1 h post-treatment **D.** The calculations based on band densitometry analysis are mentioned as applicable i.e. fold-change in the ratio of endogenous levels of CRT to Actin, relative to CNTR. Analysis for surface exposed calreticulin in non-permeabilized (treated or untreated) CT26 CO-shRNA or CRT-shRNA cells, was performed at 1 h post-treatment time-point, such that the data is presented as mean fluorescence intensity (MFI) (*n* = 3; mean ± s.d.; **p < 0.01, ***p < 0.0001 or N.S = non-significant as indicated by bars; One-way ANOVA with Dunnett's test for comparison with respective CNTR and Bonferroni's test for comparison between other conditions). Bands for CRT and Actin in (D) for all the conditions were quantified for their integrated density followed by correlation analysis between the amount of surface exposed calreticulin **E.** and endogenous CRT levels **F.** or endogenous Actin levels **G.** (*p < 0.05; correlation coefficient and *p* value are mentioned on the graph; Student's *t*-test).

Interestingly, a recent study reported that cells with low endogenous levels of CRT show inability in exposing ecto-CRT [34]. Interestingly, immunoblotting analysis revealed that AY27 cells indeed exhibit approximately 50%–60% less endogenous CRT expression compared to the ICD-susceptible CT26 cells (Fig. 4A). In line with this, correlation analysis across different cell lines (AY27/CT26) and treatments (CNTR/MTX/Hyp-PDT), showed that a strong, positive linear correlation (*r* = 0.84) existed between the endogenous CRT levels and the ability to expose ecto-CRT (Fig. 4B) [34]. Similar correlation was not found for endogenous actin levels and ecto-CRT (*r* = −0.52), a ‘negative control’ analysis (Fig. 4C).

To further confirm the validity of the above conclusions, we decided to stably knock-down the overall CRT levels in CT26 cancer cells (via shRNA), to levels approximately similar to AY27 cells. Reducing overall CRT levels in CT26 cells by 50%–60% (Fig. 4D) significantly compromised the ability of these cells to present ecto-CRT after MTX or Hyp-PDT treatment (Fig. 4E). Moreover, in line with the above analysis, correlation analysis across different CT26 clones (CO-shRNA/CRT-shRNA) and treatments (CNTR/MTX/Hyp-PDT), showed that a strong, positive linear correlation (*r* = 0.88) existed between the endogenous CRT levels and the ability to expose ecto-CRT (Fig. 4F) but not between endogenous actin levels and ecto-CRT (*r* = 0.19) (Fig. 4G). Of note, several previous studies have shown that knocking down CRT in CT26 cells compromises their ability to activate AVE [14, 30, 35, 36], an observation that we also confirmed in our CT26 CRT-shRNA set-up (data not shown).

Taken together these results show that endogenous CRT levels positively correlate with the ability of a cell to expose ecto-CRT. Moreover, AY27 cells possess a “low endogenous CRT expression” phenotype, which is probably a major factor behind their inability to present ecto-CRT.

### A subset of patients, belonging to various cancer-types, exhibit low tumoural expression levels of calreticulin

**Figure 5:**
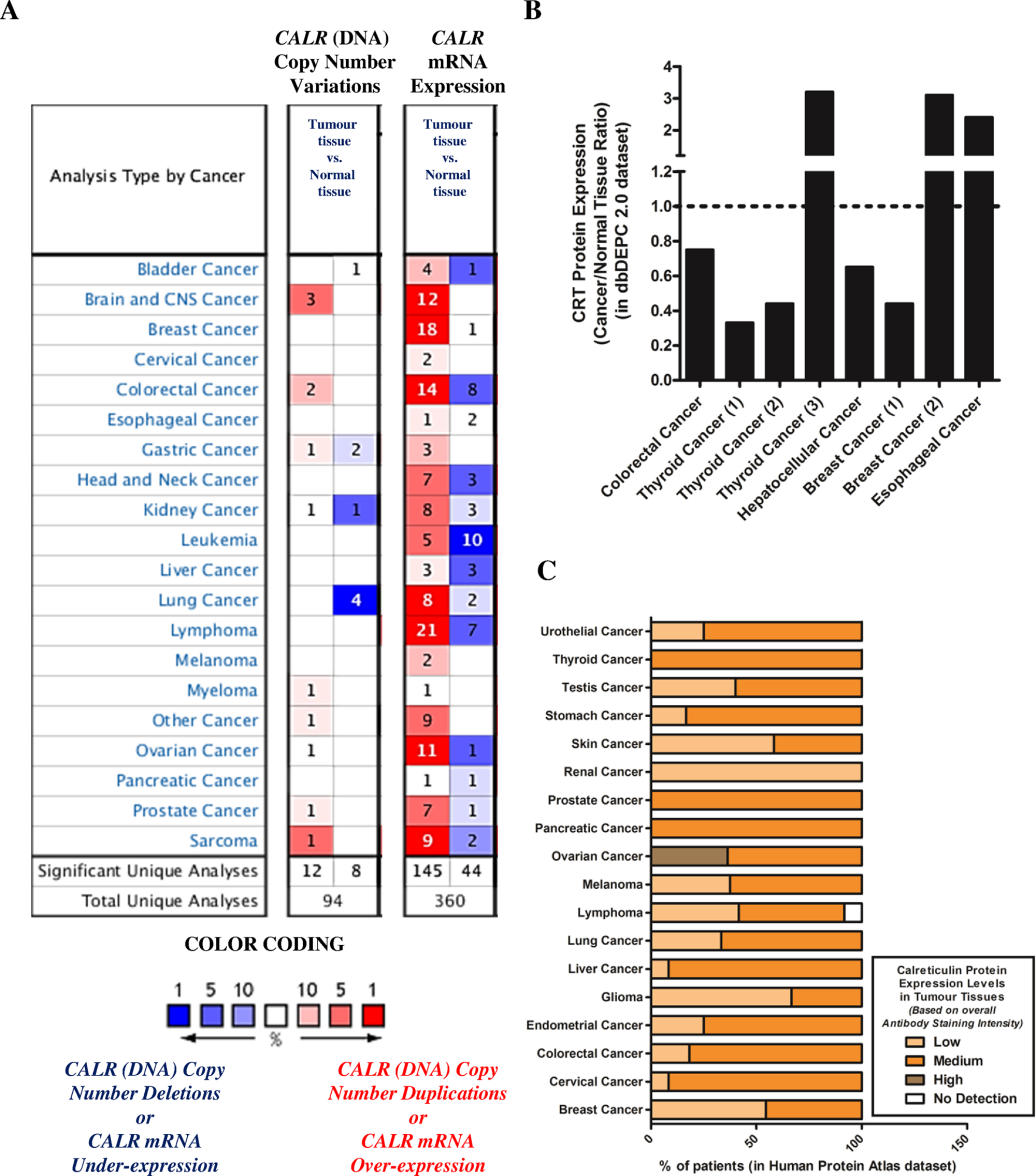
A subset of patients, of various cancer types, show low or reduced overall endogenous levels of CRT/*CALR* **A.** Differential *CALR* gene copy numbers or *CALR* mRNA levels between tissues derived from various cancer types and corresponding normal tissues were analysed using the Oncomine database (*p*-value threshold was set at less than 0.01; all fold changes were deemed valid). Over-expression or under-expression in the top 1, 5 and 10% are color-coded according to the legend. **B.** Ratio of differential CRT protein levels between tissues derived from various cancer types and corresponding normal tissues were analysed using the dbDEPC 2.0 proteomics database. **C.** Overall CRT protein levels determined in human tumour tissues via tissue microarray analysis-based immunohistochemistry were retrieved using the Human Protein Atlas database. Here, the level or overall intensity of antibody-based staining has been used to generate three annotated protein expression patterns i.e. high, medium, and low, and no detection levels (colour coded here, in the legend, where the intensity of colour indicates the level of expression/staining).

Based on our above preclinical findings, indicating that low endogenous levels of CRT could be a crucial cell autonomous factor compromising AVE potential; we set out to determine whether, a subset of patients of various cancer types could show reduced or low tumoural expression levels of CRT/*CALR*. Firstly, we carried out a data-mining exercise of publicly available DNA sequencing or microarray datasets in the Oncomine database [37]. We compared *CALR* levels between tumour tissue and corresponding normal tissue (of the same tissue-type), in order to obtain Oncomine profiles/analyses with significant *CALR* over/under-expression ratios for each combination, which are shown in respective boxes [38]. Of note, each analysis represents a logical grouping of samples based on standardized sample facts [38]. Through this analysis we found that a subset of patients of various cancer types have a tendency to show either *CALR* gene copy-number deletions (8 out of 94 total analyses; especially in kidney cancer and lung cancer) or, more importantly, *CALR* mRNA under-expression (44 out of 360 total analyses; especially in bladder cancer, colorectal cancer, head and neck cancer, leukaemia, liver cancer, lymphoma, ovarian cancer and sarcoma) in tumoural tissues relative to corresponding normal tissues (Fig. 5A).

Next, we decided to confirm whether this phenotype i.e. tumoural *CALR*^low^ is also “phenocopied” at the protein level (i.e. tumoural CRT^low^). A data-mining exercise of publicly available mass spectrometry-based proteomics datasets carried out in the dbDEPC database [39] found that certain cohorts of patients for various cancer-types have a tendency to show down-regulation of CRT protein expression i.e. tumoural CRT^low^ (between 0.20–0.60 fold less expression than corresponding normal tissue in case of thyroid cancer, hepatocellular cancer and breast cancer) (Fig. 5B). Of note, the coverage of dbDEPC in terms of number of cancer-types is much more limited than Oncomine owing to user-driven, curated submission model. In order to increase this coverage, we decided to analyse the publicly available tissue-microarray based immunohistochemistry datasets in the Human Protein Atlas database [40]. We found that there was indeed a tendency for a certain subset of patients, for various different cancer-types, to show a tumoural CRT^low^ phenotype (especially, skin cancer, renal cancer, glioma and breast cancer) (Fig. 5C).

Taken together, our analysis of publicly available DNA sequencing, microarray, proteomics and tissue-microarray datasets, showed that at least a subset of patients of various different cancer-types show low tumoural expression of *CALR*/CRT.

### Tumoural *CALR* levels predict the clinical efficacy of immunogenic cell death-inducing anticancer therapies in lung or ovarian cancer patients

**Figure 6:**
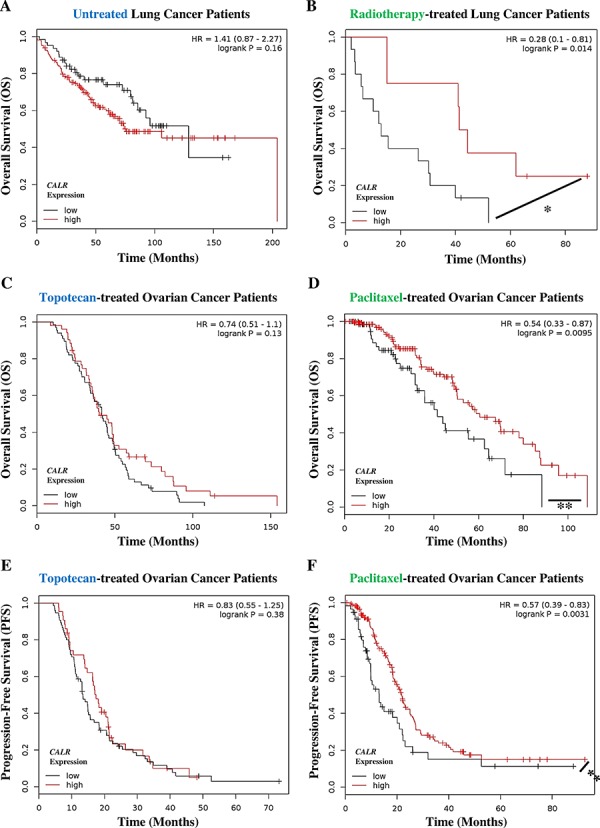
Cancer patients exhibiting low tumoural *CALR* levels show poor clinical prognosis in response to paclitaxel and radiotherapy **A, B.** Lung cancer patients not treated with any therapy (i.e. untreated, *n* = 227) (A) or treated with radiotherapy only (*n* = 23) (B) were stratified into high (red lines) or low (black lines) expression-based “risk-groups” by considering the median of the overall transcript-expressions of *CALR* (untreated – low *n* = 63, high *n* = 164; radiotherapy – low *n* = 15, high *n* = 8). This was followed by Kaplan-Meier plotting of the patient's overall survival (OS) (Y-axis). **C–D.** Ovarian cancer patients treated with topotecan only (*n* = 119) (C) or treated with paclitaxel only (*n* = 220) (D) were stratified into high (red lines) or low (black lines) expression-based “risk-groups” by considering the median of the overall transcript-expressions of *CALR* (topotecan – low *n* = 67, high *n* = 52; paclitaxel – low *n* = 69, high *n* = 151). This was followed by Kaplan-Meier plotting of the patient's OS (Y-axis). **E–F.** Ovarian cancer patients treated with topotecan only (*a* = 118) (E) or treated with paclitaxel only (*n* = 229) (F) were stratified into high (red lines) or low (black lines) expression-based “risk-groups” by considering the median of the overall transcript-expressions of *CALR* (topotecan – low *n* = 75, high *n* = 43; paclitaxel – low *n* = 57, high *n* = 172). This was followed by Kaplan-Meier plotting of the patient's progression-free survival (PFS) (Y-axis). In all the above graphs, respective log-rank test *p*-values and hazard ratios (HR; with its 95% confidence interval in parenthesis) are displayed. Statistical significance (i.e. *p* < 0.05 or *p* < 0.001) is indicated through an asterisk (* or **).

On the basis of above results, we set out to determine whether *CALR* expression could predict the tendency of a subset of patients with non-small cell lung cancer (hereafter refer to as lung cancer) or ovarian cancer to respond to ICD-inducers but not non-ICD inducers. Of note, reliable publicly available datasets for such analysis were not available for Hyp-PDT and MTX treatment however such datasets were available for radiotherapy (for lung cancer), paclitaxel and topotecan (for ovarian cancer); and hence analysis proceeded with respective patients left untreated or treated with these latter ICD inducers and non-ICD inducer. In a setting of untreated lung cancer patients, the differential tumoural *CALR* expression levels (Suppl. Fig. S4A) were not significantly relevant in defining the overall survival (OS) of patients. However, untreated patients with *CALR*^low^ tumours tended to show better OS (HR = 1.41, 95% CI 0.87–2.27) albeit this trend was not significant (Fig. 6A). On the other hand, lung cancer patients treated with the ICD inducer radiotherapy and having *CALR*^high^ tumours (Suppl. Fig. S4B), tended to have significantly better OS than radiotherapy-treated patients with *CALR*^low^ tumours (*HR* = 0.28, 95% CI 0.1–0.81) (Fig. 6B). Moreover, in ovarian cancer patients treated with the non-ICD inducer topotecan the differential tumoural *CALR* expression levels (Suppl. Fig. S4C, S4E) were not significant in defining either the OS (Fig. 6C) or progression-free survival (PFS) (Fig. 6E) of these patients. In stark contrast, ovarian cancer patients treated with the ICD inducer paclitaxel and having *CALR*^high^ tumours (Suppl. Fig. S4D, S4F), exhibited significantly better OS (Fig. 6D) as well as significantly better PFS (Fig. 6F) than paclitaxel treated patients with *CALR*^low^ tumours (for OS: HR = 0.54, 95% CI 0.33–0.87; for PFS: *HR* = 0.57, 95% CI 0.39–0.83).

**Figure 7:**
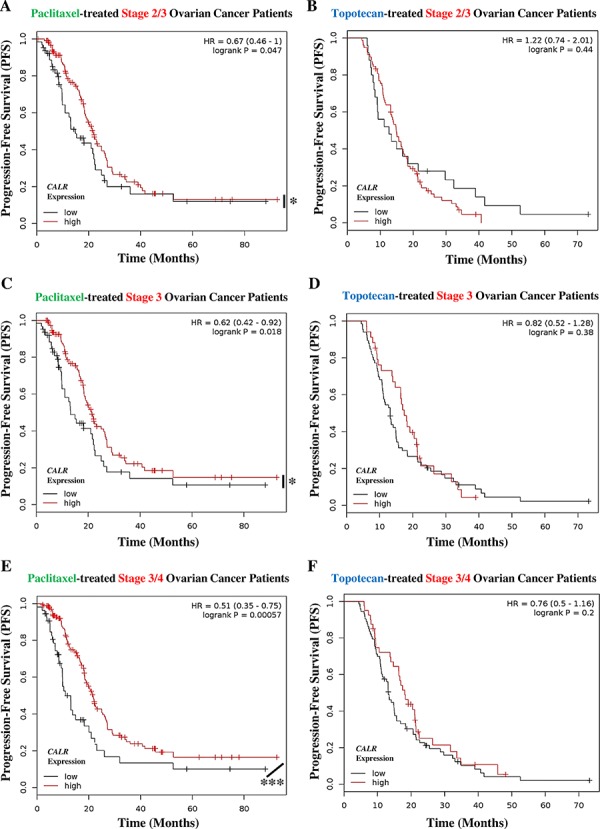
Paclitaxel treated cancer patients with tumoural *CALR^low^* phenotype, show cancer stage-independent poor clinical prognosis **A, B.** Stage 2/3 ovarian cancer patients treated with paclitaxel (*n* = 188) (A) or topotecan (*n* = 103) (B) were stratified into high (red lines) or low (black lines) expression-based “risk-groups” by considering the median of the overall transcript-expressions of *CALR* (paclitaxel – low *n* = 64, high *n* = 124; topotecan – low *n* = 25, high *n* = 78). This was followed by Kaplan-Meier plotting of the patient's progression-free survival (PFS) (Y-axis). **C–D.** Stage 3 ovarian cancer patients treated with paclitaxel (*n* = 177) (C) or topotecan (*n* = 100) (D) were stratified into high (red lines) or low (black lines) expression-based “risk-groups” by considering the median of the overall transcript-expressions of *CALR* (paclitaxel – low *n* = 62, high *n* = 115; topotecan – low *n* = 66, high *n* = 34). This was followed by Kaplan-Meier plotting of the patient's PFS (Y-axis). **E–F.** Stage 3/4 ovarian cancer patients treated with paclitaxel (*n* = 217) (E) or treated with topotecan (*n* = 113) (F) were stratified into high (red lines) or low (black lines) expression-based “risk-groups” by considering the median of the overall transcript-expressions of *CALR* (paclitaxel – low *n* = 54, high *n* = 163; topotecan – low *n* = 73, high *n* = 40). This was followed by Kaplan-Meier plotting of the patient's PFS (Y-axis). In all the above graphs, respective log-rank test *p*-values and hazard ratios (HR; with its 95% confidence interval in parenthesis) are displayed. Statistical significance (i.e. *p* < 0.05 or *p* < 0.0001) is indicated through an asterisk (* or ***).

Next we evaluated the predictive value of *CALR* expression when the patients are categorized into different cohorts according to the different tumour stages. Since there were not enough radiotherapy-treated lung cancer patients available to allow for such categorization in an objective manner, we confined this analysis to ovarian cancer. Interestingly, all the patient cohorts based on different stages of ovarian cancer showed similar trend i.e. Stage 2/3 (Fig. 7A), Stage 3 (Fig. 7C) and Stage 3/4 (Fig. 7E) ovarian cancer patients treated with the ICD inducer paclitaxel and having *CALR*^high^ tumours, exhibited significantly better PFS than paclitaxel-treated patients with *CALR*^low^ tumours (Fig. 7A, 7C, 7E). Very interestingly, this positive association between *CALR*^high^ tumours and paclitaxel treatment, became stronger and more statistically significant as the ovarian cancer stages advanced further (for Stage 2/3: HR = 0.67, 95% CI 0.46–1.00, *p*-value = 0.047; for Stage 3: HR = 0.62, 95% CI 0.42–0.92, *p*-value = 0.018; and for Stage 3/4: HR = 0.51, 95% CI 0.35–0.75, *p*-value = 0.00057) (Fig. 7A, 7C, 7E). On the other hand, the stage of ovarian cancer under consideration did not change the inability of tumoural *CALR* expression levels in predicting the patient responses to topotecan (Fig. 7B, 7D, 7F).

Together, the above results suggest that high tumoural *CALR* expression predisposes patients with lung and ovarian cancer to improved clinical responses following radiotherapy or paclitaxel-based anticancer therapy.

### Tumoural *CALR* levels positively correlate with levels of phagocytosis-related genes specifically in cancer patients treated with immunogenic cell death-inducing anticancer therapy

**Figure 8:**
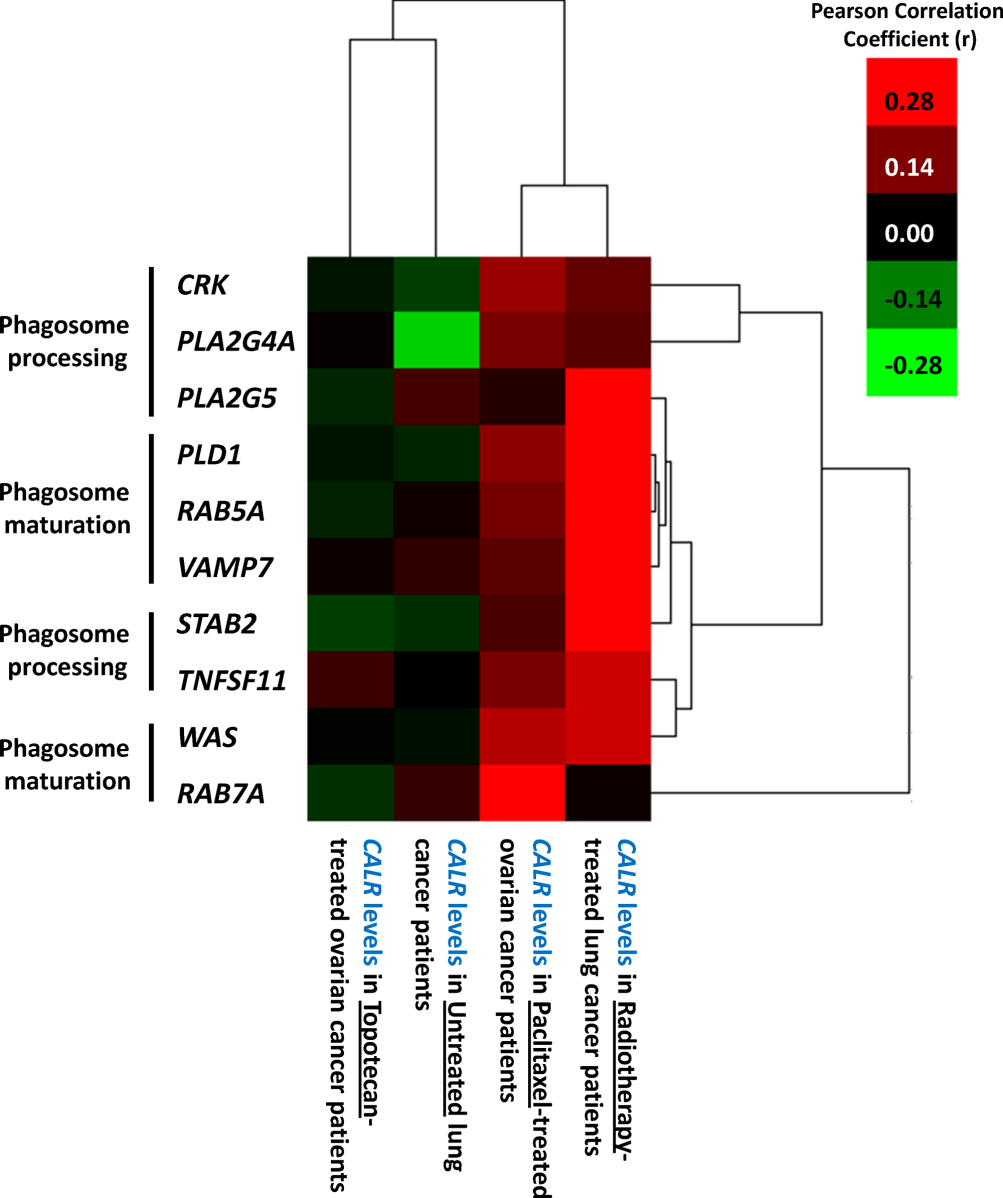
Overall tumoural *CALR* levels positively correlate with expression levels of phagocytosis-related genes only in cancer patients treated with ICD-inducing therapies Generation of gene co-expression profiles was accomplished by correlating the expression profiles of phagocytosis-related genes (*CRK*, *PLA2G4A*, *PLA2G5*, *PLD1*, *RAB5A*, *VAMP7*, *STAB2*, *TNFSF11*, *WAS*, *RAB7A*) with *CALR* expression levels for the respective scenarios indicated in the Fig. and Pearson's correlation coefficient (r) was used for indicating tendency to co-express. Thereafter the correlation profiles were clustered and represented through a heat map. The colour code is represented as legend.

Taken together, our experimental data suggested that low endogenous CRT levels leads to reduced ecto-CRT, which in turn impairs phagocytic removal and AVE inducing potential, in ICD settings. In clinical terms, we then wondered whether tumoural *CALR* levels in cancer patients treated with ICD inducers (e.g. radiotherapy-treated lung or paclitaxel-treated ovarian, cancer patients) or not (untreated lung or topotecan-treated ovarian, cancer patients) had a direct association with levels of phagocytosis-related genes. Phagocytosis-related genes utilized for this co-expression analysis included those involved in phagosome maturation (*PLD1* [41], *RAB5A* [42], *RAB7A* [43], *VAMP7* [44], *WAS* [45]) and phagosome processing (*CRK* [46], *PLA2G4A* [46], *PLA2G5* [47], *STAB2* [46], *TNFSF11* [48]) in order to get as close as possible to active phagocytosis process rather than mere presence of phagocytes in the tumour (Fig. 8). Interestingly, this correlation analysis showed that mainly in the ICD clinical settings tumoural *CALR* levels positively correlated with the tumoural levels of phagocytosis-associated genes (especially *PLA2G5*, *PLD1*, *RAB5A*, *VAMP7*, *STAB2*) (Fig. 8). On the other hand, in non-ICD settings, the tumoural *CALR* levels either didn't or even negatively correlated with the tumoural levels of phagocytosis-associated genes (Fig. 8). Moreover, within the ICD clinical setting, this positive correlation between *CALR* and phagocytosis-associated genes was higher for radiotherapy-treated lung cancer patients than for paclitaxel-treated ovarian cancer patients (Fig. 8).

In conclusion, tumoural *CALR* levels tend to positively correlate with levels of tumoural phagocytosis-associated genes, only in the ICD clinical settings.

## DISCUSSION

**Figure 9:**
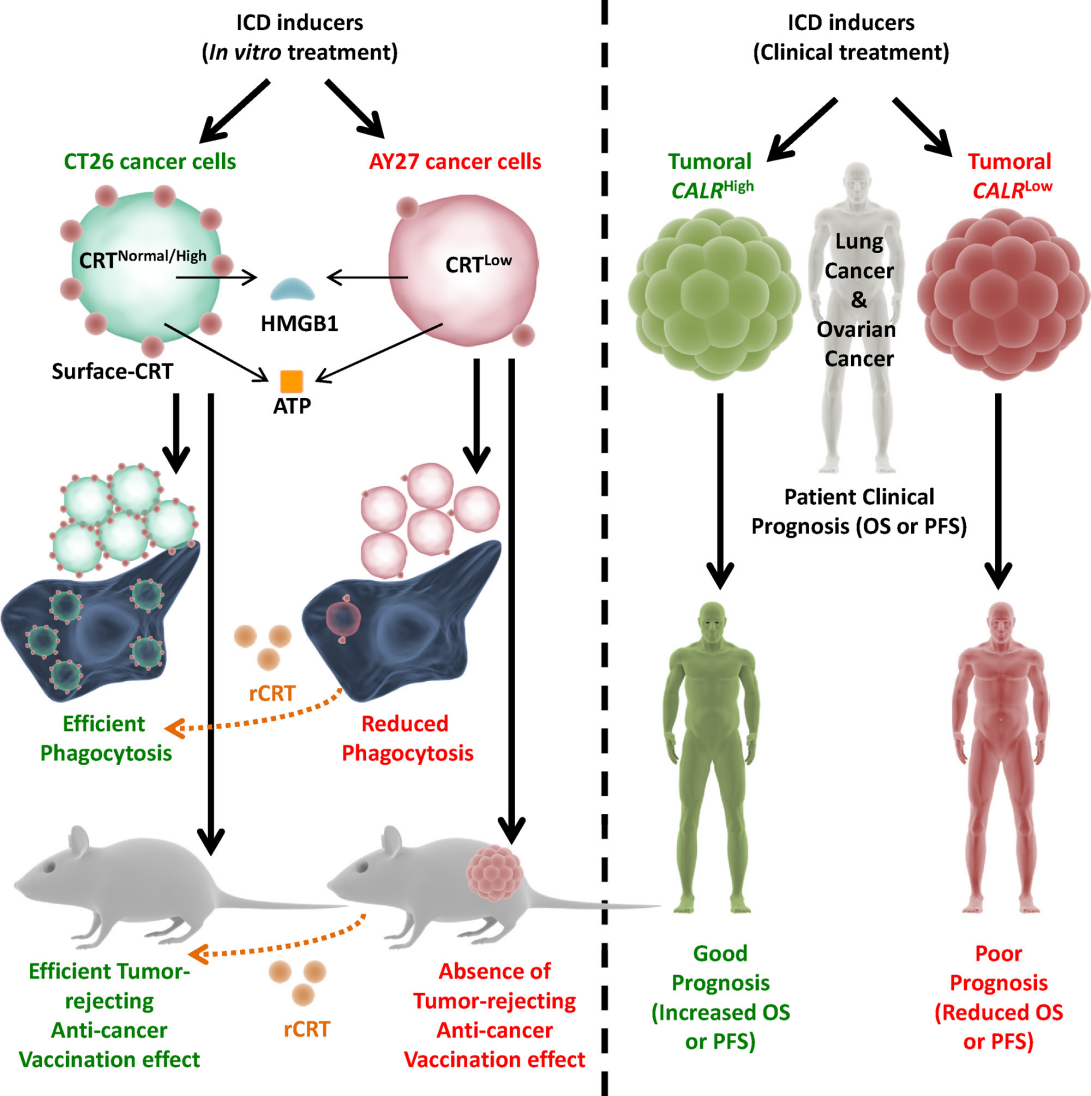
Overall endogenous CRT levels within cancer/tumoural cells dictate the *in vivo* anti-cancer vaccination effect and clinical efficacy of ICD-inducing anticancer therapies Cancer cells possessing normal or high CRT levels (e.g. CT26), when treated with ICD inducing therapies, undergo efficient phagocytosis by phagocytes and activate potent anti-cancer vaccination effect (AVE). On the other hand, cancer cells possessing low CRT levels (e.g. AY27), when treated with ICD inducing therapies, undergo inefficient phagocytosis by phagocytes and are incapable of activating AVE. However, exogenous “reconstitution” of ecto-CRT through addition of recombinant-CRT (rCRT) increases the efficiency of phagocytic clearance and increases AVE-activating capacity of the latter cancer cells. In a similar manner, lung or ovarian cancer patients treated with ICD inducers (like radiotherapy or paclitaxel) and having *CALR*^high^ tumours, tend to exhibit good clinical prognosis. On the contrary, lung or ovarian cancer patients treated with ICD inducers and having *CALR*^low^ tumours, tend to exhibit poor clinical prognosis. ATP – adenosine triphosphate, CALR – calreticulin mRNA/transcript, CRT – calreticulin protein, HMGB1 – high mobility group box 1, OS – overall survival, PFS – progression-free survival.

In the current study we characterized, for the first time, a naturally-occurring cancer cell-model that exhibits intrinsic resistance towards anticancer vaccination (Fig. 9). We went onto characterize that the reason behind this is a defect in ecto-CRT-based immunogenicity, which makes these cells resistant to ICD-induced AVE (Fig. 9). Primary (if not the only) reason behind this disparity in AY27 cells seemed to be the relatively reduced endogenous protein levels of CRT (Fig. 9). Moreover, these interesting preclinical findings and the existence of tumours displaying lower CRT expression were also supported by the observation that a subset of patients of various cancer-types presented *CALR*^low^ or CRT^low^ tumours (Fig. 9). Even more importantly, we went onto demonstrate that *CALR* expression levels were pivotally predictive of patient clinical responses to ICD-inducing anticancer therapies – an observation with possibly vital clinical implications (Fig. 9). In fact we also observed that, only in the ICD clinical setting, tumoural *CALR* levels positively correlated with the levels of phagocytosis-associated genes relevant for phagosome maturation/processing. This is a novel observation, which at least partially substantiates the pre-clinically well-established role of CRT (i.e. ecto-CRT) in mediating cancer cell phagocytosis, in clinical samples. Although, a more targeted analysis of phagocytosis in clinical tumour tissue samples via immunohistochemistry is required to further validate these results comprehensively.

AVE-resistance mechanisms represent major stumbling blocks for anticancer immunotherapy [4, 49]. Thus, in our opinion, approaches striving to characterize intrinsic ICD/AVE ‘resistant phenotypes’ are important for future research [1]. For instance, a similar approach taken on the host immune system-level, led to the discovery that naturally-occurring *Tlr4* mutation in C3H/HeJ mice caused failure of ICD/AVE – an observation that paved way for eventual characterization of a subset of breast cancer patients possessing *TLR4* SNP polymorphism (+896A/G → Asp299Gly) that associated with poor prognosis under treatment with ICD inducers [50, 51]. This, taken together with the complex and multifactorial nature of anticancer vaccination responses [1, 2], reveals that several as-yet-uncharacterized experimental models with intrinsic resistance to ICD/AVE might exist [52]. Our study advocates the systematic identification of such models since these can help explain the failure of certain subset of patients in responding to ICD-inducers, as demonstrated here.

Remarkably, except for ecto-CRT, AY27 cells emitted all the other important DAMPs recently shown to be crucial for AVE like secreted ATP and released HMGB1 [7, 8] (Fig. 9). This shows the pivotal position occupied by ecto-CRT at the interface between the cancer cells and the host immune system [7, 8]. Moreover, these observations prove that amongst this constellation of ICD-associated DAMPs, ecto-CRT is probably by far the most important DAMP – a point suggested partially by other studies from our [6, 12, 24] as well as other laboratories [11, 14, 53, 54]. For instance, melphalan-treated B78 melanoma cells used as “vaccines” were found to exhibit reduced AVE responses despite the ability of these cells to secrete ATP and present ecto-HSP90 –a defect that could only be corrected by exogenous addition of rCRT [32]. All this advocates the integration of rCRT in future anticancer vaccination paradigms.

The defective CRT protein expression-level in AY27 cells observed in this study, is also corroborated by another study showing that *in vivo*, AY27 tumours display reduced ability to modulate *CALR* expression [55]. Moreover, the AY27 cells also exhibited a relatively heightened ER stress response, a phenomenon that could be a consequence of reduction in endogenous CRT protein levels [56]. The mechanism that “created” the CRT^low^ phenotype in AY27 cells is not immediately clear. Notably, a recent study documented an immune-surveillance mechanism that tends to counter-select for cancer cells with reduced capacity to expose ecto-CRT [29]. Moreover, certain post-translational (e.g. sumoylation) or epigenetic mechanisms (e.g. microRNA or long non-coding RNA) have also been contextually characterized to increase or decrease CRT expression-levels [34, 57, 58]. Whether such mechanisms are applicable to AY27 cells is a matter of speculation and needs further systematic investigation in future. Of note, although addition of ecto-rCRT increased the phagocytosis of AY27 cells and their AVE-inducing capacity yet these increases were still relatively partial in comparison to CT26 cell model. This could be because other as-yet-unknown DAMPs or immunostimulators might also be disrupted in AY27 cells – a hypothesis that needs urgent verification in near future. This hypothesis though receives some support from an independent study showing that the *in vivo* markers of immunogenicity (e.g. increased infiltration of CD3^+^ or CD45RA^+^ lymphocytes) are reduced within AY27 tumours compared to the peri-tumoural normal tissue [55].

In clinical terms, our observation of *CALR* expression levels being predictive of clinical responses to ICD inducers like radiotherapy/paclitaxel (Fig. 9) but not to untreated or non-ICD inducer treated patients is fascinating – owing to the recent contradictory evidences surrounding the validity of *CALR* levels as predictive or prognostic biomarker. It has been observed that based on the study under consideration, high *CALR*/CRT expression level could have either positive [59, 60] or negative [53, 61] prognostic impact, or, either robust [62] or non-discernible [63] predictive effect. We shed further light on this subject by showing that tumoural *CALR* expression is in fact a context-dependent predictive biomarker of anticancer therapy response in cancer patients. Such that, while tumoural *CALR*^high^ is predictive of positive therapy responses in patients treated with ICD inducers, yet differential *CALR* expression is not predictive of responses in untreated or non-ICD inducer treated patients. Importantly, a combination of tumoural *CALR*^high^ phenotype and application of ICD inducing therapies is what dictates better patient survival. Moreover, we also provide the first glimpses that tumoural *CALR* levels might regulate phagocytosis in tumours, only in ICD but not non-ICD clinical settings. These findings may have important clinical implications that need to be further elaborated. It would be also important to examine whether tumoural *CALR* expression levels could be exploited for clinical stratification or decision-making strategies regarding the type of anticancer therapy to be applied for patient treatment.

## MATERIALS AND METHODS

### Materials and reagents

Hypericin was prepared/purified/stored as described in the past [64]. Tauroursodeoxycholic acid (TUDCA) was purchased from Merck-Millipore (Darmstadt, Germany) while Z-Val-Ala-Asp(OMe)-fmk (zVAD-fmk) was purchased from Bachem (Weil am Rhein, Germany). Mitoxantrone (MTX) and Anti-actin antibody was purchased from Sigma (St. Louis, MO, USA). Topotecan and paclitaxel were kind gifts from Prof. Johan Swinnen/Niamat Ali Khan (KULeuven, Belgium). Antibodies against CHOP/P-eIF2α/Total eIF2α/cleaved caspase 3/PARP/HSP90/Bip/GRP78 were purchased from Cell Signaling Technology (Danvers, MA, USA). Anti-calreticulin antibody (for immunoblotting) was purchased from Stressgen (Victoria, BC, Canada). Antibody against HMGB1 was purchased from Abcam (Cambridge, UK). Anti-HSP70 antibody was purchased from Santa Cruz Antibodies (San Francisco, USA). Secondary antibodies conjugated to horseradish peroxidase were purchased from Cell Signaling Technology (Danvers, MA, USA)/Abcam (Cambridge, UK). Also, the following secondary antibodies were used: goat anti-mouse-DyLight680/goat anti-rabbit-DyLight800 (Thermo Scientific, Belgium).

### Cell lines and induction of immunogenic cell death

CT26, AY27, J774 and RMW cells were cultured at 37°C under 5% CO_2_ in DMEM containing 4.5 g/L glucose and 0.11 g/L sodium pyruvate and supplemented with 2 mM glutamine, 100 units/ml penicillin, 100 μg/L streptomycin and 10% fetal bovine serum. For induction of immunogenic cell death, the cells were cultured in the presence of mitoxantrone, radiotherapy or paclitaxel (concentrations and incubation timings are mentioned in respective figure legends). For Hyp-PDT the cells were incubated with 150 nM Hypericin (for 16 h) followed by light irradiation (2.70 J/cm^2^) performed as described previously [65].

### Generation of *CALR*-shRNA stable clones of CT26 cells

All the TRC1 shRNA clones were in lentiviral pLKO.1-puro vector (Sigma-Aldrich) and were obtained from the BCCM/LMBP Plasmid collection, Department of Biomedical Molecular Biology, Ghent University, Belgium (http://bccm.belspo.be/about/lmbp.php). An empty pLKO.1-puro control vector was used as a control (CO-shRNA) (BCCM/LMBP Plasmid collection). Three shRNAs targeted against murine *CALR* mRNA were used i.e. *CALR* shRNA-1: CCGGGAGTGTAAGAACTACAAACAACTCG AGTTGTTTGTAGTTCTTACACTCTTTTT (Clone ID NM_007591.2–1823s1c1), *CALR* shRNA-2: CCGGGCTGGGTCGAATCCAAACATACTCGAGTA TGTTTGGATTCGACCCAGCTTTTT (Clone ID NM_007591.2–176s1c1) and *CALR* shRNA-3: CCGGGCAAATATCTATGCCTATGATCTCGAG ATCATAGGCATAGATATTTGCTTTTT (Clone ID NM_007591.2–976s1c1). Briefly, to generate lentivirus particles, HEK 293T cells were seeded in 25 cm^2^ flasks at 1.3 × 10^6^ cells per 4 ml and transfected the following day by the calcium phosphate method with 4 μg of pLKO.1-puro carrying the respective shRNAs or with empty pLKO.1-puro. Each transfection also included 1.2 μg of a plasmid encoding VSV-G (pMD2-VSV-G, Tronolab) and 2.6 μg of a plasmid encoding packaging proteins (pCMVdR8.9, Tronolab). For more detailed protocol please refer to Garg et al. *EMBO J* (2012) [6]. Knockdown of CRT was confirmed by immunoblotting and for further analysis only that clone was selected which showed a 50–60% knock-down of CRT (i.e. clone expressing shRNA-3).

### Anticancer vaccination experiments

All mice or rats were maintained in pathogen-free conditions and the experiments were performed according to the guidelines of the local Ethics Committee of the KU Leuven or the UGent. CT26/AY27 cells were incubated with either MTX or exposed to Hyp-PDT. After a 24 h recovery, 3 × 10^6^ of these cells were injected subcutaneously (s.c.) in 200 μl of PBS into the left flank of 7–8 weeks-old female BALB/c mice (in case of CT26 cells) from Janvier (Bio Services BV, The Netherlands) or 150–175 g female Fischer 344 rats (in case of AY27 cells) from Charles River Laboratories (Lyon, France). Control mice/rats were injected with 200 μl PBS only. Mice/rats were then challenged with 5 × 10^5^ live untreated CT26/AY27 cells in the other (right) flank 7-days after immunization. The ‘communication’ of the tumour antigenic-memory to the adaptive immune system was studied by scoring tumours' incidence at the challenge site (*i.e*. in the right flank). For this, the mice/rats were monitored every 5–6 days (post-challenge) for the presence of tumours and the experiment was stopped shortly before the tumours in control mice/rats could reach either 10% of the total mice weight or a volume of 2 cm^3^ (about 20–50 days). In some cases as applicable, the treated cancer cells were pre-coated with rCRT (Abcam) by incubating the cells with 3 μg/10^6^ cells rCRT for 30 min and washed thereafter, before injection.

### *In vitro* phagocytosis assay

Cancer cells were treated as described above and recovered after 1 h. CT26 cells, AY27 cells, J774 phagocytes and RMW phagocytes were detached with TrypLE™ Express (Life Technologies, Carlsbad, CA, USA). J774/RMW phagocytes were labelled with CellVue^®^ NIR780 and CT26/AY27 cells with CellVue^®^ Jade (eBioscience, Vienna, Austria). Respective cancer cells and phagocytes (CT26:J774 and AY27:RMW) were then incubated at a 1:5 ratio for 4 h. Cells were then harvested using TrypLE™ Express and analysed on flow cytometer (Attune, Life Technologies, Carlsbad, CA, USA). The percentage of cancer cells that were phagocytosed was calculated by dividing the number of double-positive cell number by the number of the JADE positive cells. In some cases as applicable, the treated cancer cells were pre-coated with rCRT by incubating the cells with 3 μg/10^6^ cells rCRT (Abcam) for 30 min and washed thereafter, before co-incubation with phagocytes.

### Immunoblotting

Cell lysate preparation for immunoblotting, protein concentration analysis and the respective immunoblotting analysis was carried out as described previously [6, 32]. The detection of signal was done using the Odyssey infrared-imaging system (Li-Cor Biosciences, Lincoln, NE, USA) or Chemidoc^TM^ MP system (Bio-Rad, Nazareth Eke, Belgium), as applicable. Densitometric analyses were generated using Image J.

### Cell viability and apoptosis/cell death analysis

Cells were seeded at 5000 cells per well of a 96-well plate. After treatment, cells were analysed via MTS assay (Promega), as per the manufacturer's instructions. Annexin-V-APC and SytoxGreen (SyGr) staining for apoptosis/cell death analysis and its FACS-based analysis was done as described previously [32].

### Measurement of damage-associated molecular patterns (DAMPs)

After treatment, cells were collected with TrypLE Express (Life Technologies, Belgium), washed with PBS and with FACS (Flow Cytometry) buffer (2% FBS, 1% BSA in PBS), incubated for 1 hr at 4°C with anti-CRT antibody (Abcam, Cambridge, UK), washed and incubated for 1 hr at 4°C with goat anti-rabbit-Alexa Fluor^®^647 (Invitrogen, Belgium). After final washes cells were incubated in FACS buffer including 1 μM Sytox Green (Life Technologies, S7020) for 15 min and analysed on Attune Flow Cytometer (Life Technologies). The permeabilised cells were excluded from the analysis, and the mean fluorescence intensity (MFIs) for ecto-CRT was analysed. On the other hand, in another case, after treatment, extracellular ATP was measured in the conditioned media using an ATP Bioluminescent assay kit (Sigma, St. Louis, MO, USA) based on luciferin-luciferase conversion, as described previously [6, 12, 24]. Bioluminescence was assessed by optical top reading *via* FlexStation 3 microplate reader (Molecular Devices Inc., Sunnyvale, CA, USA). Last but not least, for analysis of post-death released DAMPs, the ‘conditioned’ culture media (5–8 ml), were collected and concentrated to 200–500 μl *via* centrifugation (3500 × g for 25–45 min) using Pierce Concentrator 7ml/9K filters (Pierce), according to the manufacturer's instructions. They were then analysed by immunoblotting as described below.

### Clinical dataset and patient survival analysis

For characterization of differential *CALR* expression in patients, analysis was done for - DNA copy-number aberrations/mRNA expressions using the Oncomine database (http://www.oncomine.org) [37], proteomics-based protein expression analysis using the dbDEPC 2.0 database (http://lifecenter.sgst.cn/dbdepc/index.do) [39] and tissue microarray-based immunohistochemical protein-expression using the Human Protein Atlas (http://www.proteinatlas.org) [40].

For predictive biomarker analysis, the respective microarray gene expression data and clinical survival information from TCGA, EGA or GEO databases were analysed through the KMPlotter platform [66] for lung and ovarian cancer patients. The respective patients were stratified into two risk-groups i.e. patient group showing high *CALR* expression and patient group showing low *CALR* expression by considering the median expression over the entire dataset. Biased arrays were excluded. The effect of differential gene expression was estimated on the overall survival (OS) or progression-free survival (PFS) of the patients (as applicable) by plotting Kaplan-Meier curves. Hazard ratio (and its 95% confidence intervals) and logrank *P* values were calculated (*P* values less than 0.05 were considered to be statistically significant). Of note, the respective results were selected only if two out of three *CALR* microarray probes supported the same patient survival patterns and representative probes or patterns out of the final two, were selected on the basis of statistical significance (logrank *p* < 0.05). Patients surviving over the follow-up threshold were censored.

### Analysis of correlation between *CALR* levels and phagocytosis-related genes' levels

For generating gene co-expression profiles for *CALR* vs. phagocytosis-related genes in respective tumoural samples, the expression profiles of individual phagocytosis-related genes were correlated with *CALR* as applicable and Pearson's correlation coefficient (r) was used for indicating tendency to co-express. Thereafter, the respective correlation coefficient values were “pooled” and hierarchical clustering of these respective correlation-based gene co-expression profiles was implemented as described elsewhere [67] through Cluster 3.0 [68] and visualized as a heatmap through TreeView [69] with Correlation (uncentered) as the similarity metric and a centroid linkage clustering criteria.

### Statistical analysis

All statistical analyses were performed using either Prism software (GraphPad Software, USA) or GraphPad QuickCalcs online software (http://www.graphpad.com/quickcalcs/index.cfm). Student's *t*-test, Fischer's test and One-way ANOVA were used for statistical analysis, as applicable and unless otherwise mentioned, with significance level set at *p* < 0.05.

## SUPPLEMENTARY FIGURES



## Abbreviations

ATP: adenosine triphosphate
AVE: anticancer vaccination effect
CALR: calreticulin (mRNA/gene)
CRT: calreticulin (protein)
DAMP: damage-associated molecular patterns
Ecto: surface exposure
ER: endoplasmic reticulum
HMGB1: high mobility group box-1
HSP: heat shock protein
Hyp-PDT: Hypericin-based photodynamic therapy
ICD: immunogenic cell death
