# Correction: Resistance to anticancer vaccination effect is controlled by a cancer cell-autonomous phenotype that disrupts immunogenic phagocytic removal

**DOI:** 10.18632/oncotarget.25721

**Published:** 2018-06-26

**Authors:** Abhishek D. Garg, Sanne Elsen, Dmitri V. Krysko, Peter Vandenabeele, Peter de Witte, Patrizia Agostinis

**Affiliations:** ^1^ Cell Death Research & Therapy (CDRT) Unit, Department of Cellular and Molecular Medicine, KU Leuven University of Leuven, Leuven, Belgium; ^2^ Laboratory for Molecular Biodiscovery, Department of Pharmaceutical Sciences, KU Leuven, Leuven, Belgium; ^3^ Molecular Signaling and Cell Death Unit, Department for Molecular Biomedical Research, VIB, Ghent, Belgium; ^4^ Department of Biomedical Molecular Biology, Ghent University, Ghent, Belgium

**This article has been corrected:** The correct Supplementary Figure S2C is given below. The authors’ explanation is as follows:

**Supplementary Figure S2:**
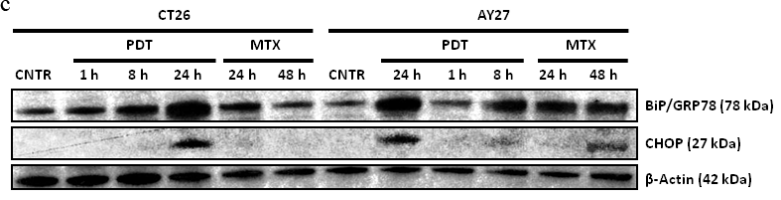
Rat bladder carcinoma AY27 cells were treated or not (i.e. untreated controls/CNTR) with Hypericin-based Photodynamic Therapy (Hyp-PDT; 150 nM Hyp incubated for 16 h followed by irradiation with light fluence of 2.70 J/cm^2^) or mitoxantrone (MTX; 1 μM) C. immunoblotting for other ER stress markers like upregulation of BiP/GRP78 or CHOP levels at indicated recovery time-points. The calculations based on band densitometry analysis are mentioned as applicable.

Due to erroneous figure processing and oversight, unintended mistakes occurred during the assembly of Supplementary Figure S2C – specifically, the BiP/Grp78, CHOP and Actin panels for the AY27-PDT samples only. Using the source/raw data, we have now generated the correct Supplementary Figure S2C.

The authors apologize for the oversight. The authors declare that this correction does not affect the description, interpretation, or the original conclusions of the manuscript.

Original article: Oncotarget. 2015; 6:26841-26860. https://doi.org/10.18632/oncotarget.4754

