# Supplementary material for: Resistance to anticancer vaccination effect is controlled by a cancer cell-autonomous phenotype that disrupts immunogenic phagocytic removal
Source: Oncotarget. 2015 Jul 23;6(29):26841–60. doi: 10.18632/oncotarget.4754 (PMC4694957; doi:10.18632/oncotarget.4754)
Supplement: Supplementary file 1 [file oncotarget-06-26841-s001.pdf]

SUPPLEMENTARY FIGURES

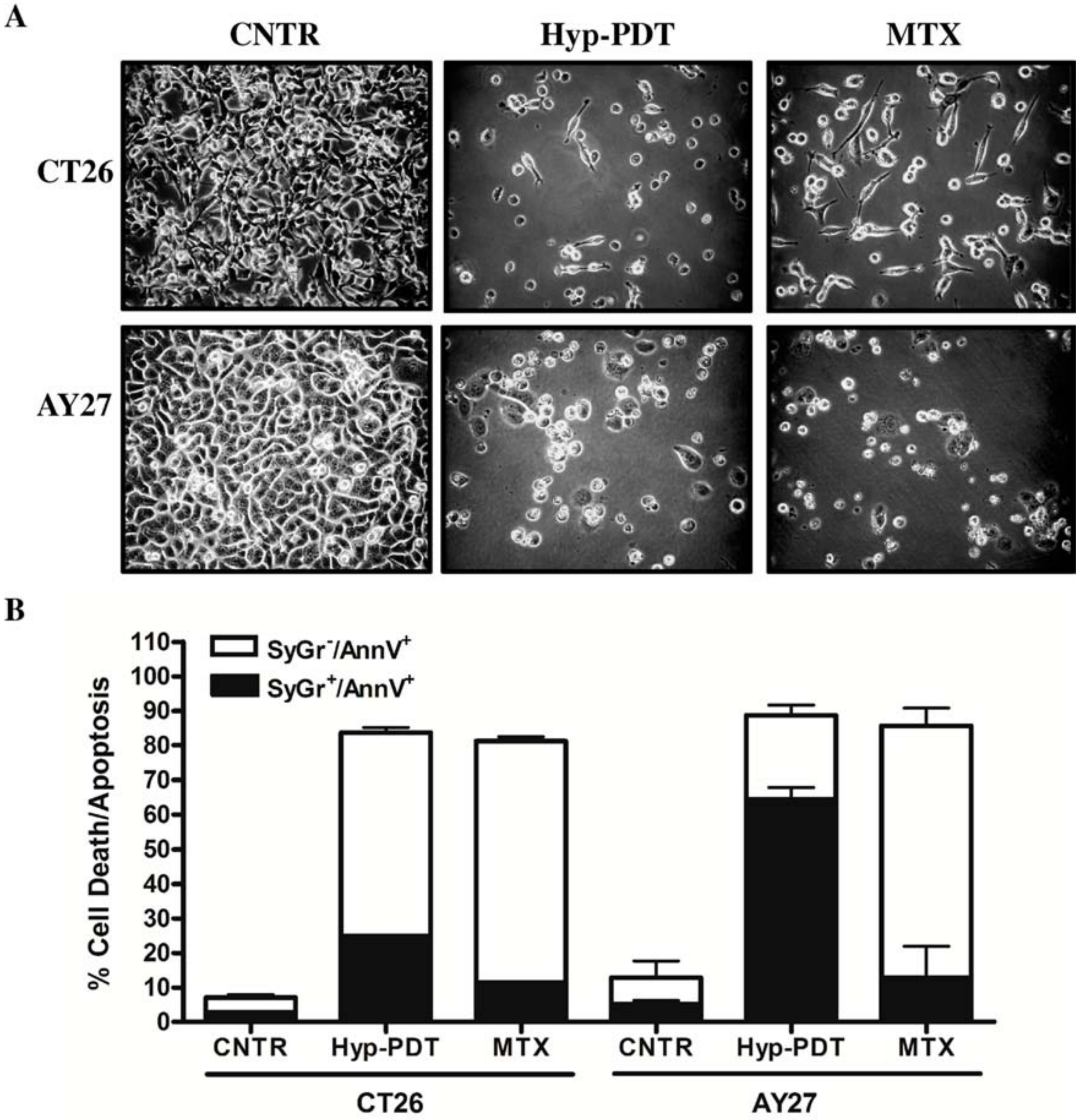

Supplementary Figure S1: Rat bladder carcinoma AY27 cells and murine colon carcinoma CT26 cells were treated or not (i.e. untreated controls/CNTR) with Hypericin-based Photodynamic Therapy (Hyp-PDT; 150 nM Hyp incubated for 16 h followed by irradiation with light fluence of 2.70 J/cm^2) or mitoxantrone (MTX; 1 µM). The cells were recovered 24 h post-treatment and either analyzed via phase-contrast microscopy **A.** or stained with Annexin V-APC and SyGr. **B.** In latter case, early apoptotic (Annexin $V^{+}SyGr^{-}$) or late apoptotic/secondary necrotic cells (Annexin $V^{+}SyGr^{+}$) were scored via FACS-based analysis ($n = 3$; means ± S.D.).

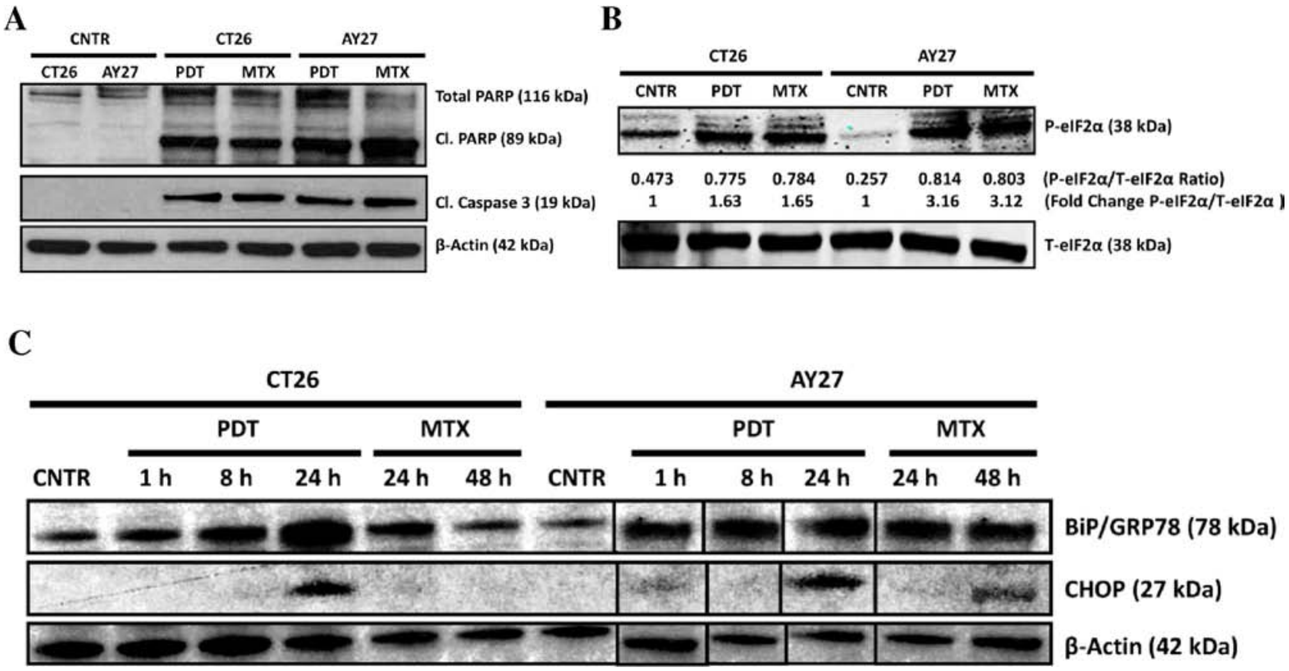

Supplementary Figure S2: Rat bladder carcinoma AY27 cells were treated or not (i.e. untreated controls/CNTR) with Hypericin-based Photodynamic Therapy (Hyp-PDT; 150 nM Hyp incubated for 16 h followed by irradiation with light fluence of 2.70 J/cm^2) or mitoxantrone (MTX; 1 µM). This was followed by - **A.** immunoblotting analysis for molecular characteristics of apoptosis i.e. detection of cleaved PARP or caspase-3, 24 h post-treatment; **B.** immunoblotting analysis for phosphorylation of eIF2α (P-eIF2α) versus total (T-eIF2α), 1 h post-treatment for Hyp-PDT and 4 h post-treatment for MTX; and **C.** immunoblotting for other ER stress markers like upregulation of BiP/GRP78 or CHOP levels at indicated recovery time-points. The calculations based on band densitometry analysis are mentioned as applicable.

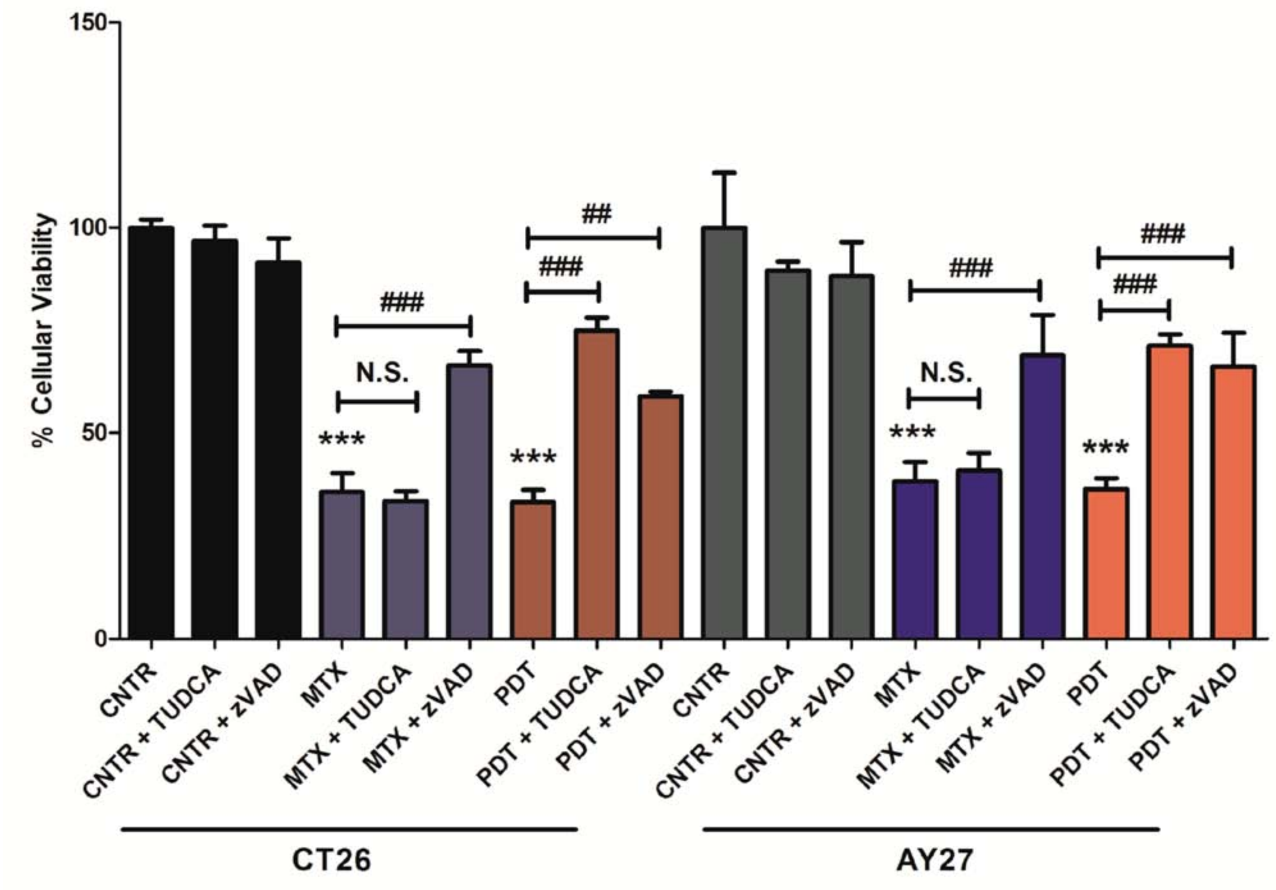

Supplementary Figure S3: AY27 cells were pre-incubated with TUDCA (500 µg/ml) or zVAD-fmk (25 µM) for 1 h. Thereafter they were treated or not (i.e. untreated controls/CNTR) with Hypericin-based Photodynamic Therapy (Hyp-PDT; 150 nM Hyp incubated for 16 h followed by irradiation with light fluence of 2.70 J/cm^2) or mitoxantrone (MTX; 1 µM). This was followed by the recovery of cells 24 h post-treatment and percentage viability of cells (i.e.% cellular viability) was assessed by MTS-based cytotoxicity assay ($n = 3$; mean ± s.e.m.; *$p < 0.05$ versus respective CNTR and #$p < 0.05$ as indicated by bars; One-way ANOVA with Bonferroni's test).

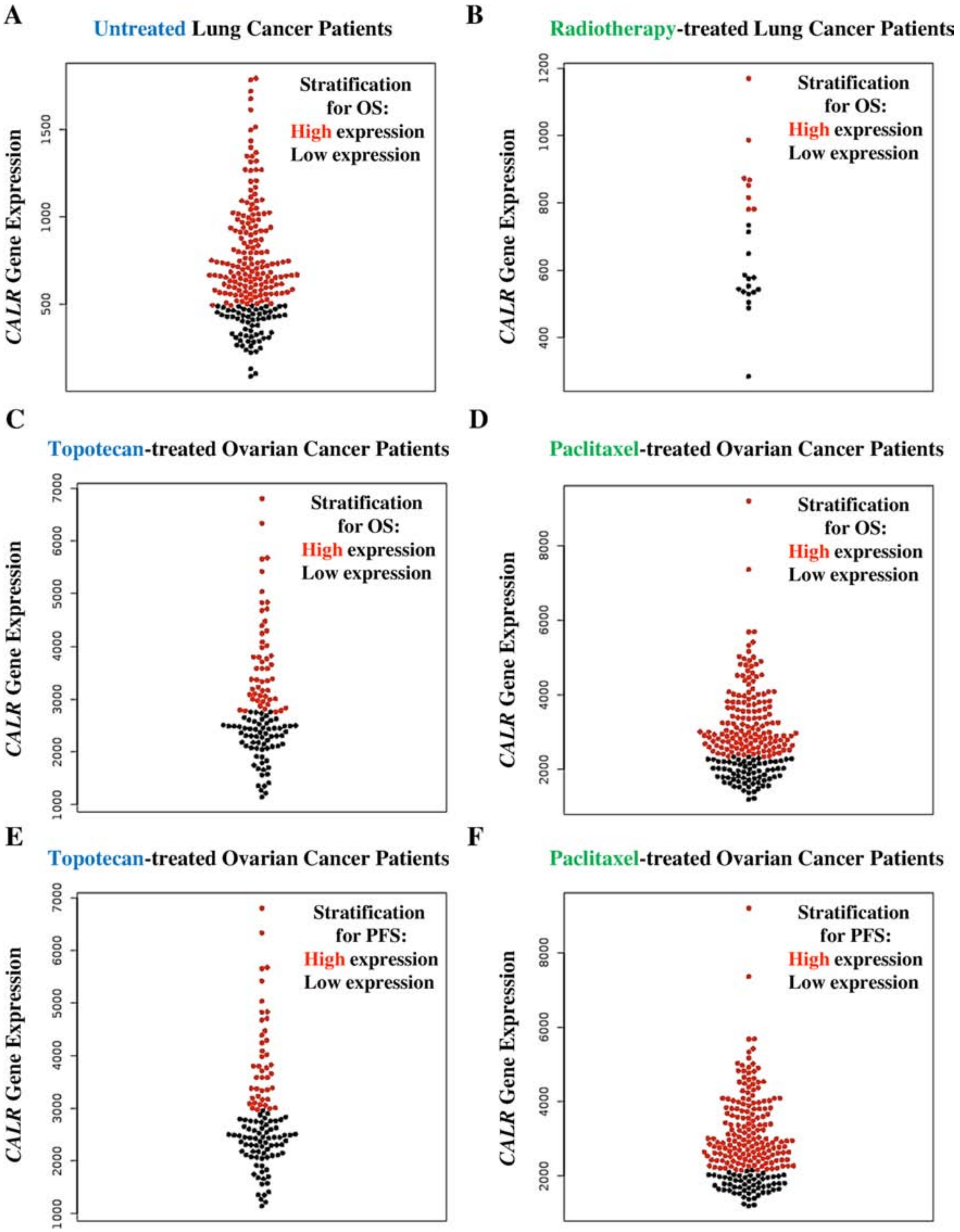

Supplementary Figure S4: Bee-swarm gene expression scatter-plots of *CALR* are shown for respective treatment or untreated conditions in lung or ovarian cancer patients, as indicated in the figure. The respective patients have been stratified into high (red dots) or low (black dots) expression profiles by considering the median of the overall transcript/mRNA-expression levels.
